# Supplementary material for: Effects of sensorimotor delays and muscle force capacity limits on the performance of feedforward and feedback control in animals of different sizes
Source: PLoS Comput Biol. 2026 Apr 21;22(4):e1012502. doi: 10.1371/journal.pcbi.1012502 (PMC13152214; doi:10.1371/journal.pcbi.1012502)
Supplement: S1 Text — In the document, we have provided detailed derivations for the equations in the paper, and described secondary analyses that we performed to evaluate our results. The document has the following sections:. S1. Delays in the feedforward and feedback pathways. S2. Bode plot analysis of a linear feedback control system. S3. Normalized feedback control system with time delays and actuator force capacity—detailed derivations and analyses. S4. Normalized feedback model predictions vs. scaled model simulation results. S5. Components of total applied torque under feedback control. S6. Comparing swing and posture task responses to in-vivo perturbation studies. (DOCX) [file pcbi.1012502.s001.docx]

# Supplementary materials

# S1. Delays in the feedforward and feedback pathways

Reflex loops suffer from several sensorimotor delays ($t_{SM}$) which are distributed across the feedforward (motor) and feedback (sensory) pathways [1]. Between the sensors (e.g. cutaneous receptors, muscle spindles) and the spinal synapse with the motor neuron, there are sensing delays, nerve conduction delays and synaptic delays—we refer to these delays together as sensory delays ($t_{SD}$). Between the spinal synapse and muscles, there are nerve conduction delays, neuromuscular junction delays, electromechanical delays and force generation delays—we refer to these delays together as motor delays ($t_{MD}$). In the feedback control model, we placed all the sensorimotor delays in the feedback (sensory) pathway to simplify the analyses (Fig 1). We could instead have placed the motor delays in the feedforward (motor) pathway, and the sensory delays in the feedback (sensory) pathway. Here, we briefly discuss how this assumption does not affect our estimates of response time, as it doesn’t affect settling times for a step response. The closed loop transfer function of a system $J\left( s \right)$, with a transfer function $G\left( s \right)$ in the feedforward (motor) pathway and $H\left( s \right)$ in the feedback (sensory) pathway is given by $\frac{G\left( s \right)}{1+G\left( s \right)H\left( s \right)}$. Therefore, the block diagram in [Fig A](#Fig_S1) has the transfer function:

$$\begin{aligned} J\left( s \right)=\frac{\theta\left( s \right)}{R\left( s \right)}=\frac{G\left( s \right)}{1+G\left( s \right)H\left( s \right)}=\frac{\left( K_{p}+K_{d}s \right) e^{-t_{MD}s} \frac{1}{Is^{2}}}{1+\left[ \left( K_{p}+K_{d}s \right) e^{-t_{MD}s} \frac{1}{Is^{2}} e^{-t_{SD}s} \right]} \#S1 \end{aligned}$$

­$\begin{aligned} \theta\left( s \right)\left[ Is^{2}+\left( K_{p}+K_{d}s \right) e^{-\left( t_{MD}+t_{SD} \right)s} \right]=R\left( s \right)\left[ \left( K_{p}+K_{d}s \right) e^{-t_{MD}s} \right] \#S2 \end{aligned}$

$$\begin{aligned} I{\theta\left( s \right) s}^{2}+K_{p} e^{-\left( t_{MD}+t_{SD} \right)s} \theta\left( s \right)+K_{d} e^{-\left( t_{MD}+t_{SD} \right)s} \theta\left( s \right) s \\ =K_{p} e^{-t_{MD}s} R\left( s \right)+K_{d} e^{-t_{MD}s} R\left( s \right) s \#S3 \end{aligned}$$

Converting Eqn S3 from the $s$ domain to the time domain:

$$\begin{aligned} I\ddot{\theta}\left( t \right)+K_{p} \theta\left( t-\left( t_{MD}+t_{SD} \right) \right)+K_{d} \dot{\theta}\left( t-\left( t_{MD}+t_{SD} \right) \right) \\ =K_{p} r\left( t-t_{MD} \right)+K_{d} \dot{r}\left( t-t_{MD} \right) \#S4 \end{aligned}$$

$$\begin{aligned} I\ddot{\theta}\left( t \right)=\tau_{act}\left( t \right) \\ =K_{p}\left[ r\left( t-t_{MD} \right)-\theta\left( t-\left( t_{MD}+t_{SD} \right) \right) \right]+K_{d}\left[ \dot{r}\left( t-t_{MD} \right)-\dot{\theta}\left( t-\left( t_{MD}+t_{SD} \right) \right) \right] \#S5 \end{aligned}$$

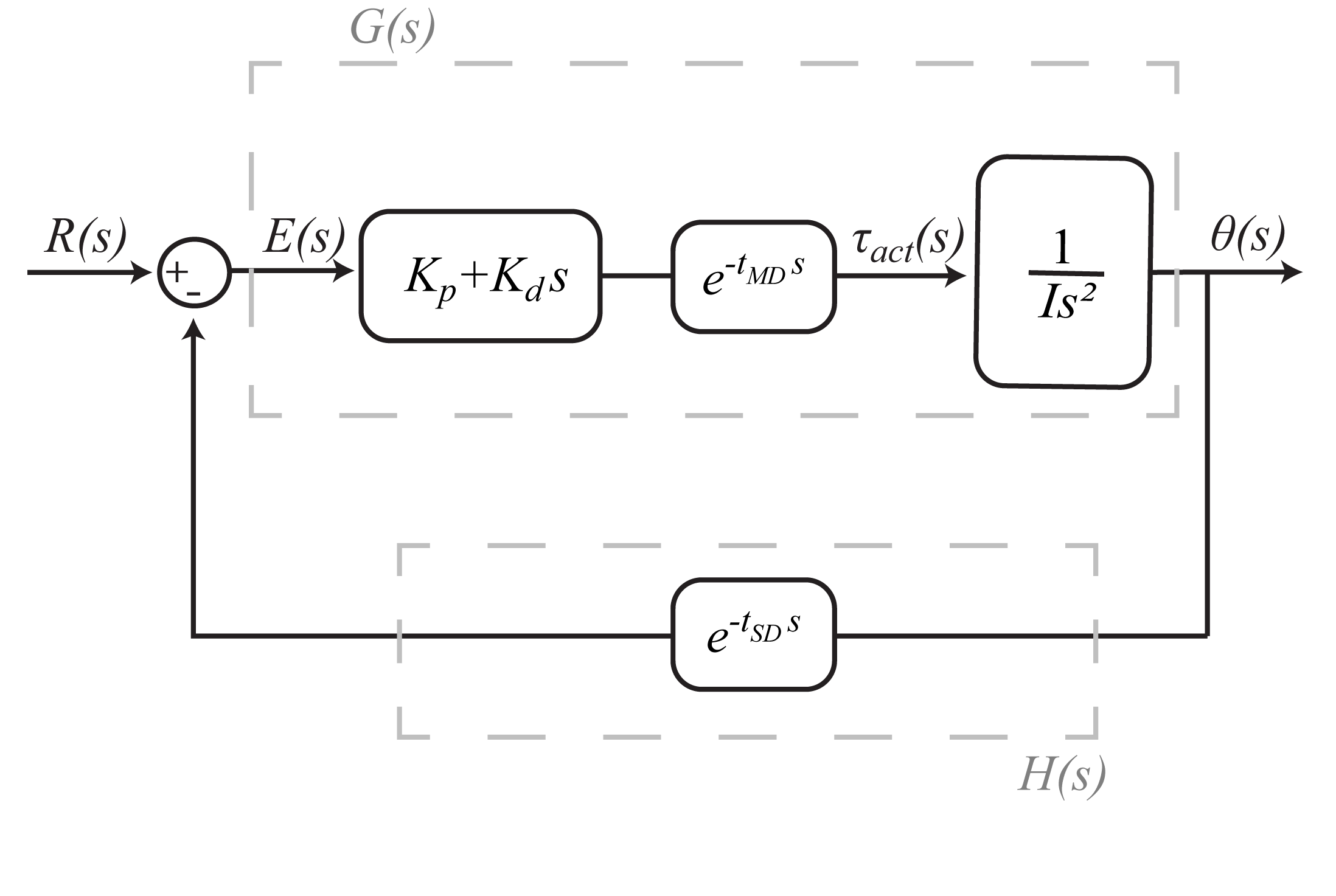


Fig A. Block diagram with delays in feedforward and feedback pathways

$R\left( s \right)$ is the reference signal, $E\left( s \right)$ is the error signal, $\theta(s)$ is the plant output (angle of the pendulum represented by the double integrator), and $\tau_{act}(s)$ is the actuating torque. $K_{p}$ and $K_{d}$ are the controller gains, and $\frac{1}{Is^{2}}$ represents the double integrator plant. $e^{-t_{MD}s}$ is the transfer function that delays the motor commands due to the time delay $t_{MD}$, and$e^{-t_{SD}s}$ is the equivalent transfer function for the sensory feedback.

The total sensorimotor delay is the sum of the sensory and motor delays:

$$\begin{aligned} t_{SM}=t_{MD}+t_{SD}\#S6 \end{aligned}$$

[Eqn S5](#Eqn_S5) demonstrates that when there are separate delays in the sensory and motor pathways, the reference signal is delayed only by motor delay ($t_{MD}$), while sensory feedback is delayed by the total delay ($t_{SM}$).

However, if we combine all the delays and put them in the feedback (sensory) pathway, we could remove $t_{MD}$ from [Eqn S5](#Eqn_S5) and replace $t_{SD}$ with $t_{SM}$ to get:

$$\begin{aligned} I\ddot{\theta}\left( t \right)=\tau_{act}\left( t \right) \\ =K_{p}\left[ r\left( t \right)-\theta\left( t-t_{SM} \right) \right]+K_{d}\left[ \dot{r}\left( t \right)-\dot{\theta}\left( t-t_{SM} \right) \right] \#S7 \end{aligned}$$

A comparison of [Eqns S5](#Eqn_S5) and S7 clarifies how having delays in both the feedforward and feedback pathways will make the tracking of a time varying reference signal more difficult vs. if there were delays only in the feedback pathway. However, since we only consider a step response in our simulations, the reference signal is constant. Therefore, [Eqn S5](#Eqn_S5) and S7 are equivalent.

# S2. Bode plot analysis of a linear feedback control system

Here, we revisit a textbook example from linear control systems theory to explain how time delays limit the controller gains that can be used in a stable feedback system. Bode plots are a frequency domain tool for linear feedback systems that determine stability margins; these margins represent the range of parameters that one can use without destabilizing the system. For this analysis, we linearized the scaled models in section 2.1 by ignoring gravity, removing time delays and force capacity limits, and simplifying the plant to a double integrator (a dynamical system representing a single degree-of-freedom rotational system). We derived and analyzed the equations using MATLAB’s symbolic toolbox. [Fig B](#Fig_S2) shows a block diagram of the linear feedback control system. It represents a Proportional-Derivative (PD) controlled double integrator, and the second-order differential equation of this system is given by:

$$\begin{aligned} I\ddot{\theta}\left( t \right)=\tau_{act}\left( t \right)=K_{p}\left[ r(t)-\theta(t) \right]+K_{d}\left[ \dot{r}\left( t \right)-\dot{\theta}(t) \right]\#S8 \end{aligned}$$

where *I* is the moment of inertia of the plant, and $K_{p}$ and $K_{d}$ are the controller gains. Note that $r(t)$ represents the sinusoidal reference signals used for Bode plots, which is different from
$\theta_{r}$ used in the scaled models (the constant target angle for the perturbation response). $\ddot{\theta}, \dot{\theta} and \theta$ are the acceleration, angular velocity and angle of the plant, respectively. The controller produces torques $\tau_{act}$ to reduce the error between the reference signal and the current state, $e\left( t \right)=r\left( t \right)-\theta\left( t \right).$

We performed a Laplace transform on Eqn S8 to convert it from the time domain to the frequency domain, e.g. $r(t)\to R(s)$, in order to get a transfer function that maps the commanded frequency$R\left( s \right)$ onto the plant output $\theta(s)$ . The open loop transfer function of the control system is given by:

$$\begin{aligned} G(s)=\frac{K_{d}s+K_{p}}{{Is}^{2}} \#S9 \end{aligned}$$

The closed loop transfer function of the control system with unity feedback is given by:

$$\begin{aligned} H(s)=\frac{G\left( s \right)}{1+G\left( s \right)}=\frac{K_{d}s{+K}_{p}}{{Is}^{2}+K_{d}s+K_{p}} \#S10 \end{aligned}$$

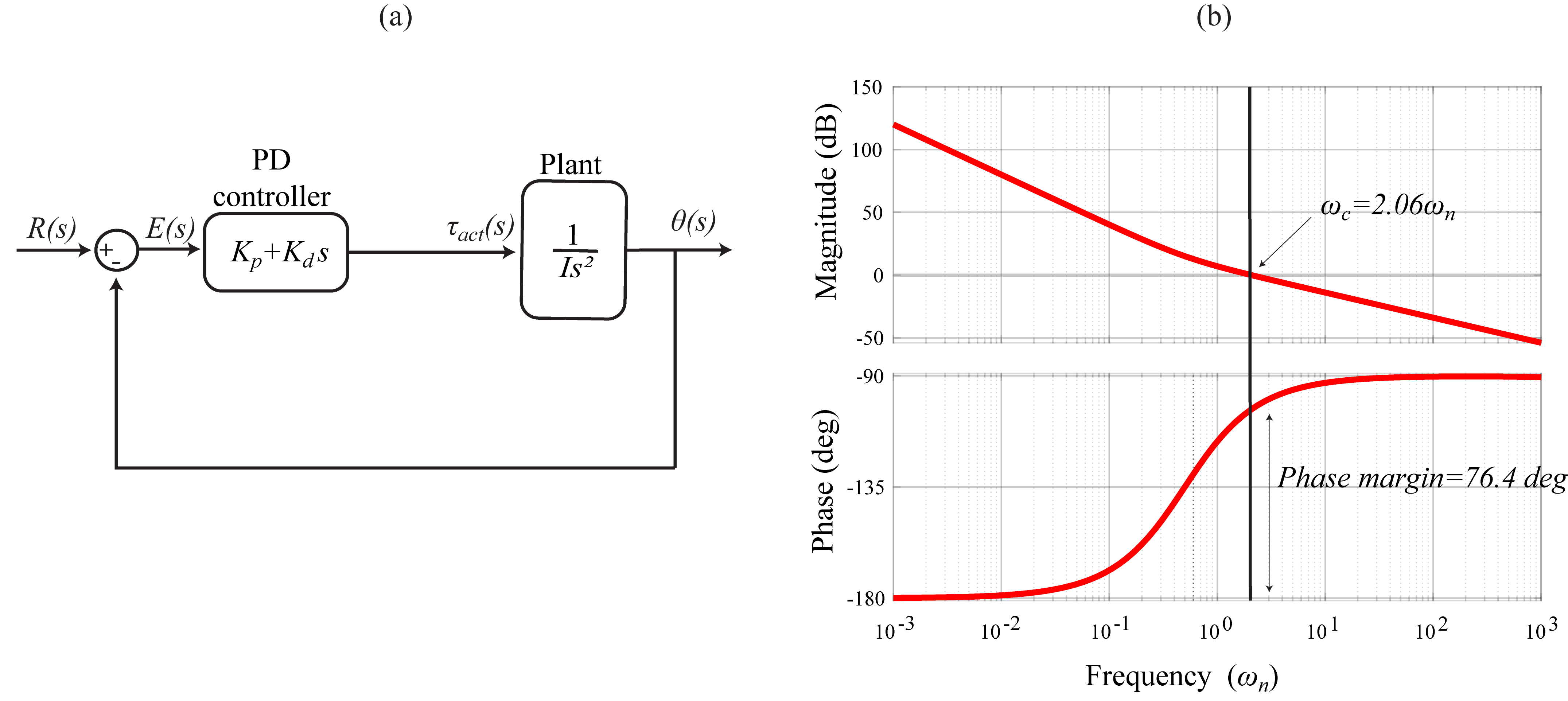


Fig B. Linear feedback control system and Bode plot

(a) The block diagram of the linear control system, where $R\left( s \right)$ is the reference signal, $E\left( s \right)$ is the error signal, $\theta(s)$ is the plant output (angle of the plant represented by the double integrator), $\tau_{act}(s)$ the actuating torque, and $s$ indicates that these time-dependent variables are represented in the frequency domain. (b) Bode plot of the open loop transfer function [(Eqn S9)](#Eqn_S9) with the gain plot on top and the phase plot on the bottom. We also show the gain crossover frequency [(Eqn S12)](#Eqn_S12) and phase margin [(Eqn S14)](#Eqn_S14) on the plot.

The equation for this closed loop control system is equivalent to that for a mechanical mass-spring-damper system. We derived two more parameters which describe the behavior of the system—the undamped natural frequency $\left( \omega_{n}=\sqrt{\frac{K_{p}}{I}} \right)$ and the damping ratio $\left( \zeta=\frac{K_{d}}{2\sqrt{K_{p} I}} \right)$ [2–4]. In order to simplify the system, we assumed that the moment of inertia ($I$) is constant, and that the system is critically damped. A critically damped system has a damping ratio of 1 $\left( \begin{matrix} \zeta=1, & K_{d}=2\sqrt{K_{p} I} \end{matrix} \right)$, and produces the fastest response without any overshoot [4]. While the scaled models had nine parameters ($I,M, g, L, K_{p}, K_{d}, \tau_{iso}, t_{SM}, \theta_{r}$), the linear system initially had three ($I, K_{p}, K_{d}$), and we have further reduced the system to just a single free parameter ($K_{p}$).

A Bode plot of the open loop transfer function provides the stability margins of the control system [(Fig Bb)](#Fig_S2). If the open loop transfer function $G(s)$equals -1, the closed loop transfer function in [Eqn S10](#Eqn_S10) becomes ∞, signifying instability. This occurs when $G(s)$ has a magnitude of 0 dB and a phase of -180° for any input frequency in the Bode plot. In this condition, the feedback signal will actively amplify the error and destabilize the feedback control system, instead of attenuating the error and stabilizing it. The phase margin is the distance of the phase line [(Fig Bb bottom)](#Fig_S2) from -180°, at the gain crossover frequency ($\omega_{c}$) — the frequency where the gain line crosses the 0 dB line [(Fig Bb top)](#Fig_S2). We found the gain crossover frequency by solving for the frequency at which the gain of the open loop transfer function equaled 1 (0 dB).

$$\begin{aligned} \left| G(s) \right|=\left| \frac{K_{p}+K_{d}\left( j\omega_{c} \right)}{{I\left( j\omega_{c} \right)}^{2}} \right|=1\#S11 \end{aligned}$$

Substituting in the value of $K_{d}$ for critical damping and solving for $\omega_{c}$ gives:

$$\begin{aligned} \omega_{c}=\sqrt{\sqrt{5}+2} \sqrt{\frac{K_{p}}{I}}=2.06 \omega_{n}\#S12 \end{aligned}$$

The phase margin is how far the phase of the open loop transfer function at the gain crossover frequency is from 180°:

$$\begin{aligned} \emptyset\left( G(s) \right)=\tan^{-1} \left( \frac{K_{d}\omega_{c}}{K_{p}} \right)-\tan^{-1} \left( \frac{0}{-I {\omega_{c}}^{2}} \right)=-1.81 rad\#S13 \end{aligned}$$

Eqn S13 reduces to a constant value because substituting in Eqn S12 for $\omega_{c}$ and the critical damping equation for $K_{d}$ causes all variable terms to cancel out.

$$\begin{aligned} Phase margin=\pi-1.81=1.33 rad (76.4 ^{\circ})\#S14 \end{aligned}$$

If this feedback system is subjected to a phase shift that exceeds the phase margin, it will become unstable. Time delays in the signal pathways can cause a phase shift, and the time delay that corresponds to the phase margin is called the delay margin. We obtained the delay margin by dividing the phase margin by the gain crossover frequency:

$$\begin{aligned} Delay margin=\frac{1.33}{2.06 \omega_{n}}= 0.647\sqrt{\frac{I}{K_{p}}}\#S15 \end{aligned}$$

As animals have a fixed sensorimotor time delay within their reflex pathways ($t_{SM}$), we can invert Eqn S15 to show that the maximum proportional gain ${K_{p}}_{max}$ that a control system with a given time delay $t_{SM}$ can use is:

$$\begin{aligned} {K_{p}}_{max}={0.647}^{2}\frac{I}{{t_{SM}}^{2}}\#S16 \end{aligned}$$

At ${K_{p}}_{max}$, we can also determine $K_{d}$ and $\omega_{n}$:

$$\begin{aligned} \begin{matrix} K_{d}=2 \left( 0.647\frac{I}{t_{SM}} \right) & \omega_{n}=0.647\frac{1}{t_{SM}} \end{matrix} \#S17 \end{aligned}$$

Thus, for a feedback control system with time delay to remain stable, it must limit its controller gains such that:

$$\begin{aligned} {0\leq K}_{p}\leq{0.647}^{2}\frac{I}{{t_{SM}}^{2}} \#S18 \end{aligned}$$

Eqn S17 shows that the time delay will cap the maximum natural frequency of the feedback system. We also find the following associations between the parameters of the feedback system: $K_{p}\propto\frac{I}{{t_{SM}}^{2}}$, $K_{d}\propto\frac{I}{t_{SM}}$, and $\omega_{0}\propto\frac{1}{t_{SM}}$.

These associations help inform our choice of non-dimensionalization factors in the next section.

# S3. Normalized feedback control system with time delays and force capacity limits—detailed derivations and analyses

We developed a normalized feedback control system (one model which can represent animals of all sizes), and studied how response times are affected by time delays and muscle force capacity (saturation) limits [(Fig C)](#Fig_S3). To keep the paper concise, the main manuscript describes an abridged version of this analysis. Here, we have provided the detailed description and complete text of this analysis. The equations of motion of the feedback control system before normalization are:

$$\begin{aligned} I\ddot{\theta}(t)=\tau_{act}(t)\#S19 \end{aligned}$$

$$\begin{aligned} \tau_{act}=sat\left( \tau_{des} \right)=\left\{ \begin{matrix} \tau_{iso} & if \tau_{des}>\tau_{iso} \\ \tau_{des} & {if -\tau}_{iso}\leq\tau_{des}\leq\tau_{iso} \\ {-\tau}_{iso} & if \tau_{des}<{-\tau}_{iso} \end{matrix} \right. \#S20 \end{aligned}$$

$$\begin{aligned} \tau_{des}=\left\{ \begin{matrix} 0 & if 0<t<t_{SM} \\ K_{p}\left[ \theta_{r}-\theta\left( t-t_{SM} \right) \right]+K_{d}\left[ -\dot{\theta}\left( t-t_{SM} \right) \right] & if t\geq t_{SM} \end{matrix} \right.\#S21 \end{aligned}$$

where $\tau_{des}$ is the desired controller output. $\tau_{act}$ is the actual torque applied to plant, subject to force capacity limits $\tau_{iso}$. We did not consider actuator (muscle) dynamics in our models, and directly applied the controller output as torques to the plant. We also assumed that the controller gets full state information; we did not consider sensory dynamics. Due to time delays $t_{SM}$, there is an initial deadtime at the start of the simulation where no torque is applied to the plant (Eqn S21). We only consider a constant reference target $\theta_{r}$ in these simulations.

We normalized Eqns S20 and S21 using three constants which also represent characteristic features of the neural control of movement in animals:

$I$ is the moment of inertia of the double integrator plant, and also represents the moment of inertia of the body segments being moved under neural control by the animal.

$\theta_{r}$ is the reference target for feedback control, and also represents the size of the movement being commanded under neural control in the swing task. For the posture task, the movement size is represented by the initial perturbation velocity $\dot{\theta}_{0}$.

$t_{SM}$ is the time delay in the feedback control system, and also represents the sensorimotor delays in the reflex pathways during neural control of movement.


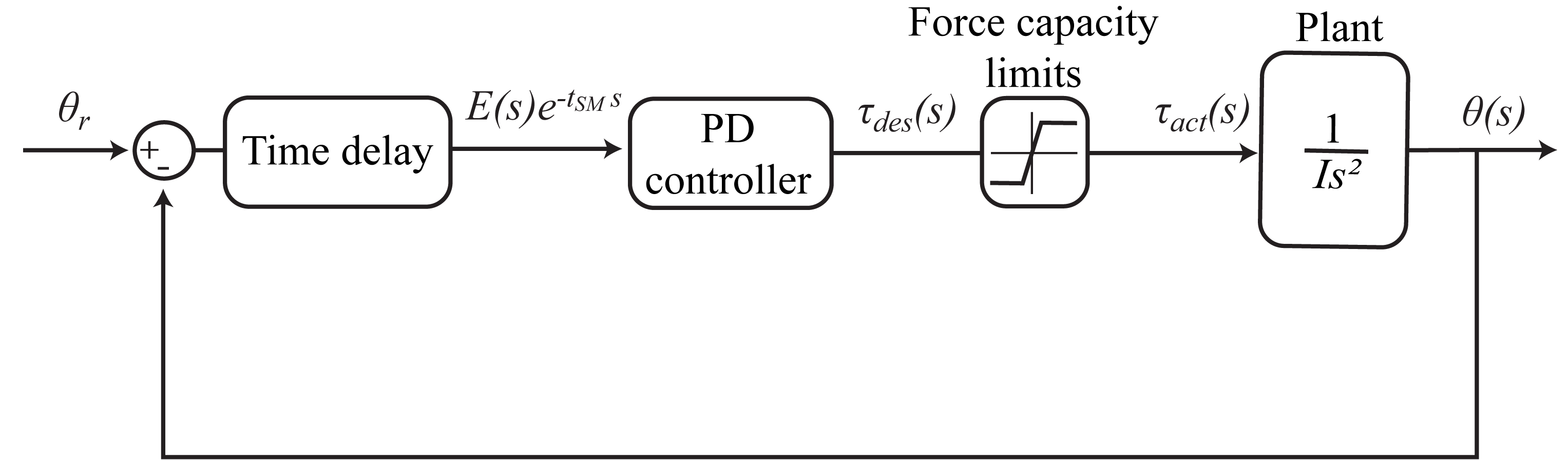


Fig C. Feedback control system with time delays and force capacity limits

$\theta_{r}$ is the reference target, $E(s)$ is the error signal, $\theta(s)$ is the plant output (angle of the pendulum represented by the double integrator), $\tau_{des}$ is the controller output torque, and $\tau_{act}$ the actuating torque subject to force capacity limits. $E(s)e^{-t_{SM} s}$ is the time delayed error signal.

The terms in [Eqn S21](#Eqn_S21) have dimensions of torque (M^1^L^2^T^-2^), where M represents mass, L represents length and T represents time dimensions. Therefore, to derive the normalized model, we divided each term in [Eqns S20](#Eqn_S20) and [S21](#Eqn_S21) by $\frac{I\theta_{r}}{{t_{SM}}^{2}}$.

$$\begin{aligned} \overline{\tau}_{des}=\left\{ \begin{matrix} 0 & if 0<\overline{t}<1 \\ \overline{K}_{p}\left[ 1-\overline{\theta}\left( \overline{t}-1 \right) \right]+\overline{K}_{d}\left[ -\dot{\overline{\theta}}\left( \overline{t}-1 \right) \right] & if \overline{t}\geq1 \end{matrix} \right.\#S22 \end{aligned}$$

$$\begin{aligned} \ddot{\overline{\theta}}=\overline{\tau}_{act}=sat\left( \overline{\tau}_{des} \right)=\left\{ \begin{matrix} \overline{\tau}_{iso} & if \overline{\tau}_{des}>\overline{\tau}_{iso} \\ \overline{\tau}_{des} & if {-\overline{\tau}}_{iso}\leq\overline{\tau}_{des}\leq\overline{\tau}_{iso} \\ {-\overline{\tau}}_{iso} & if \overline{\tau}_{des}<{-\overline{\tau}}_{iso} \end{matrix} \right. \#S23 \end{aligned}$$

The normalized model is described by Eqns S22 and S23. Below, we describe how to normalize each of the parameters of the model.

$$\begin{aligned} \begin{matrix} \overline{t}=\frac{t}{t_{SM}} & \overline{t}_{resp}=\frac{t_{resp}}{t_{SM}} \end{matrix} \#S24 \end{aligned}$$

$$\begin{aligned} \begin{matrix} \overline{\theta}=\frac{\theta}{\theta_{r}} & \dot{\overline{\theta}}=\frac{\dot{\theta}}{\left( \frac{\theta_{r}}{t_{SM}} \right)} & \ddot{\overline{\theta}}=\frac{\ddot{\theta}}{\left( \frac{\theta_{r}}{{t_{SM}}^{2}} \right)} \end{matrix} \#S25 \end{aligned}$$

$$\begin{aligned} \begin{matrix} \overline{\theta}_{0}=\frac{\theta_{0}}{\theta_{r}} & {\dot{\overline{\theta}}}_{0}=\frac{\dot{\theta}_{0}}{\left( \frac{\theta_{r}}{t_{SM}} \right)} \end{matrix} \#S26 \end{aligned}$$

$$\begin{aligned} \begin{matrix} \overline{\tau}=\frac{\tau}{\left( \frac{I\theta_{r}}{{t_{SM}}^{2}} \right)} & \overline{\tau}_{iso}=\frac{\tau_{iso}}{\left( \frac{I\theta_{r}}{{t_{SM}}^{2}} \right)} \end{matrix} \#S27 \end{aligned}$$

$$\begin{aligned} \begin{matrix} \overline{K}_{p}=\frac{K_{p}}{\left( \frac{I}{{t_{SM}}^{2}} \right)} & \overline{K}_{d}=\frac{K_{d}}{\left( \frac{I}{t_{SM}} \right)} \end{matrix} \#S28 \end{aligned}$$

$\overline{t}_{resp}$ is the normalized response time of the model, determined using numerical simulations and optimization.

## Normalized feedback control system—numerical simulations

Using numerical simulations, we evaluated how changing the perturbation task, controller gains, time delays and force capacity limits affected the behavior of the normalized feedback control system. First, we did not set force capacity limits, used a fixed time delay ($\overline{t}$=1), and performed a brute force search to determine how controller gains affect settling time and overshoot. Next, we kept time delays constant ($\overline{t}$=1), and varied the force capacity limits to understand how this affects response times ($\overline{t}_{resp}$ vs $\overline{\tau}_{iso}$). For each force capacity limit, we optimized the controller gains to find the fastest response time (fastest settling time with 2% thresholds and without any overshoot).

### Swing task results

A brute force search through a range of controller gains revealed the settling time and overshoot landscapes for the swing task. We calculated both settling time and overshoot on the angle curve for the swing task [(Fig Da bottom panel)](#Fig_S4). The settling time landscape is very rough and jagged with several local minima [(Fig Db)](#Fig_S4). The overshoot landscape has a flat area with zero overshoot, followed by a region of rapid increase [(Fig Dc)](#Fig_S4). The black dot depicts the fastest settling time achieved while allowing overshoot. The red dot depicts the fastest settling time without overshoot; we used this result to determine response time. Controller gains of 0.1617 for $\overline{K}_{p}$ and 0.6343 for $\overline{K}_{d}$ produced the fastest response time ($\overline{t}_{resp}$) of 7.09 in normalized time units [(Fig Da)](#Fig_S4).

Analysis of the relationship between force capacity limits and response time revealed three distinct regions [(Fig E)](#Fig_S5). The fastest response without considering force capacity limits produced a peak torque of 0.1617, which equals $\overline{K}_{p}$. [Fig E](#Fig_S5) shows how response time, overshoot and controller gains changed when we varied force capacity limits from 0.25 to 0.001.

- High force capacity limits region: For $\overline{\tau}_{iso}$> 0.1610, the peak torque commanded by the controller did not reach the force capacity limits, and the response time and controller gains did not change.
- Middle region: For 0.079<$\overline{\tau}_{iso}$<0.1610, the force capacity limits clipped the positive region of the torque curve, and response time increased gradually with lower force capacity limits. However, the optimal control gains still remained the same.
- Low region: 0.009<$\overline{\tau}_{iso}$<0.079, the force capacity limits clipped both the positive and negative regions of the torque curve, and response time increased exponentially with lower force capacity limits. In this region, the controller gains also increased with lower force capacity limits.


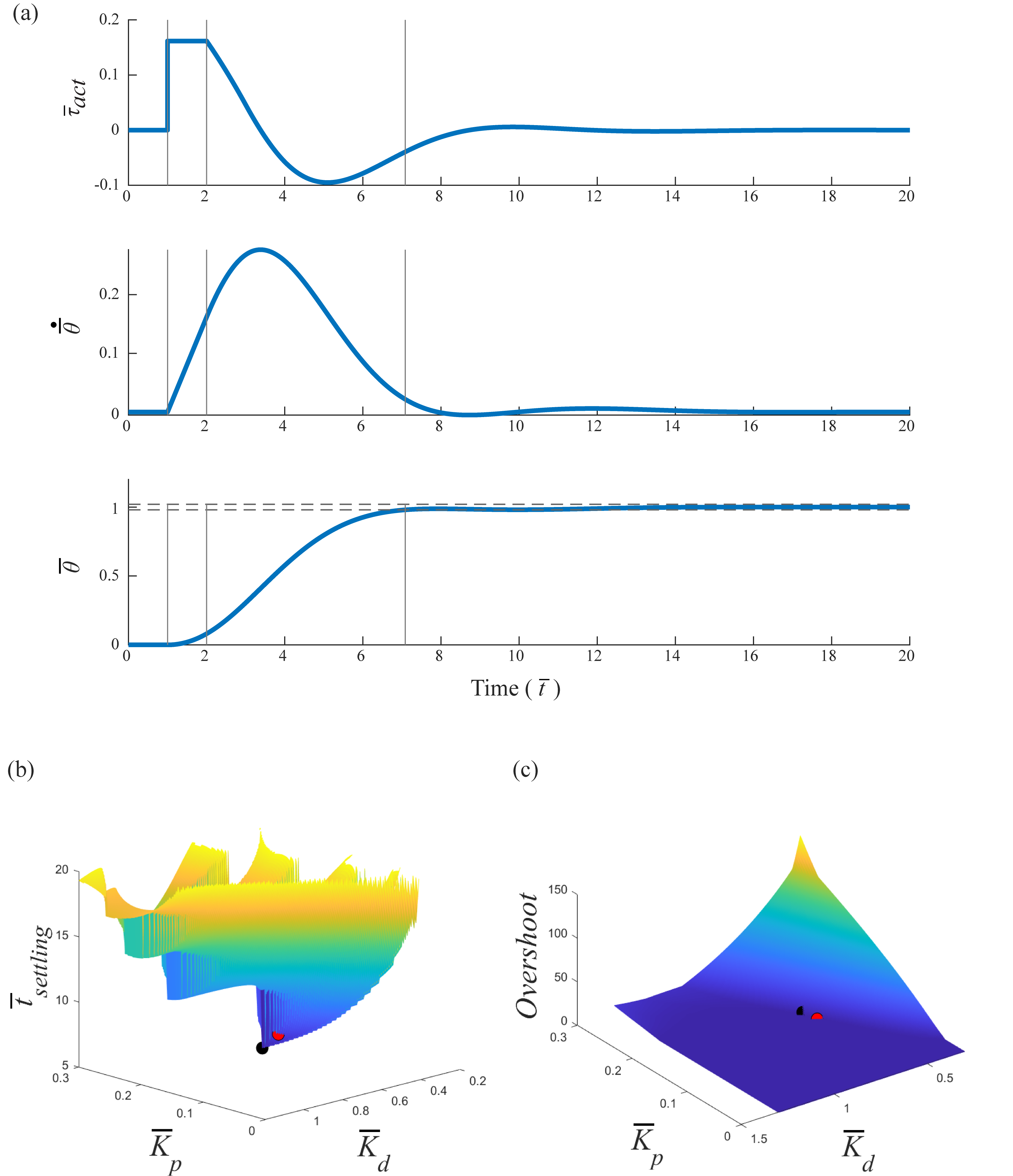


Fig D. Normalized swing task—brute force search

(a) Normalized torque ($\overline{\tau}_{act}$), angular velocity ($\dot{\overline{\theta}}$) and angle ($\overline{\theta}$) profiles in the swing task for the fastest response. The three grey vertical lines depict one time delay period, two time delay periods and the settling time. The two grey horizontal dashed lines on the angle graph depict the 2% settling time thresholds. (b & c) The settling time and overshoot landscapes for the normalized swing task model determined through a brute force search for a range of controller gains. The black dot depicts the fastest settling time when overshoot is allowed (This response had 1.76% overshoot). The red dot depicts the result we use for response time—the fastest settling time without overshoot.


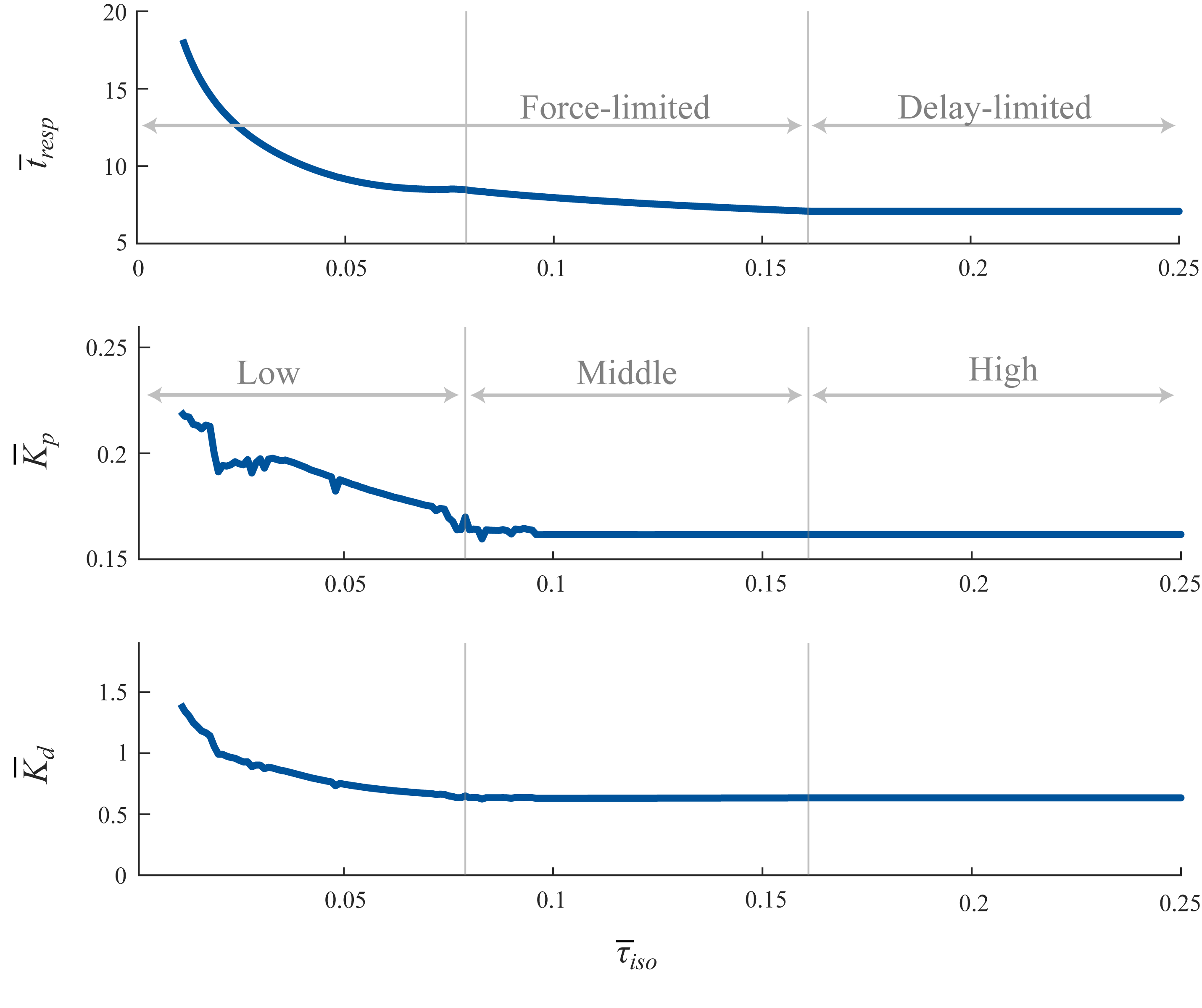


Fig E (Fig 2). Normalized swing task—force capacity limits vs. response time

(a) Changes in response time and overshoot when force capacity limits are lowered from 0.25 to 0. There are three distinct regions in the response time profile, shown divided by the grey vertical lines. (b) Changes in the optimal controller gains that produce the fastest response times without overshoot for a range of force capacity limits. Note that there is some variability in the optimal controller gains in the middle and low regions due to multiple local minima very close to the global minimum, but they do not affect the response time significantly.

To evaluate whether the normalized model can predict response times in the more detailed scaled model simulations, we used curve fitting to estimate the relationship between normalized force capacity and normalized response time ($\overline{t}_{resp}$ vs. $\overline{\tau}_{iso}$). We found that the double exponential function gave the best fit (lowest root mean square error) for the middle and low force capacity limit regions in the swing task. The swing task $\overline{t}_{resp}$ vs. $\overline{\tau}_{iso}$ relationship can be described by the following function:

$$\begin{aligned} \overline{t}_{resp}=f\left( \overline{\tau}_{iso} \right)=\left\{ \begin{matrix} 7.09 & \overline{\tau}_{iso}\geq0.162 \\ 5.56e^{-27.1\overline{\tau}_{iso}}+8.64e^{-1.29\overline{\tau}_{iso}} & {0.079\leq\overline{\tau}}_{iso}<0.161 \\ 18.33e^{-73.12\overline{\tau}_{iso}}+10.12e^{-2.88\overline{\tau}_{iso}} & {0.009\leq\overline{\tau}}_{iso}<0.078 \end{matrix} \right. \#S29 \end{aligned}$$

This analysis shows that for feedback control systems with both time delays and force capacity limits, there are two ranges in the response time vs. force capacity limits graph: a force-limited range and a delay-limited range. At the extremes, without force capacity limits or delays, infinitely high gains can produce instant response times. If we reduced the force capacity limits to 0, or if the time delays were infinitely long, we would have infinite response times. The delay-limited range matches the high force capacity limits region ($\overline{\tau}_{iso}$ >0.1617), where the response time is limited purely by time delays. The force-limited range consists of the middle and low force capacity limits regions ($\overline{\tau}_{iso}$ <0.1617), where the force capacity limits also begin to limit response time. Extrapolating to animals, this analysis indicates that for an animal to respond quickly, it requires both strong muscles and short sensorimotor delays. Deficiencies in either factor will slow the animal’s ability to respond quickly. Whether an animal is delay-limited or force-limited would depend on the relative magnitudes of factors that affect the perturbation response, such as the moment of inertia of the body segments being moved, the sensorimotor delays, the size of the perturbation response movement, and the muscle force capacity.

### Posture task results

For the normalized posture task model, the brute force search results were similar to the swing task, while the relationship between response time and force capacity limits had several differences. For the posture task, we calculated settling time on the angular velocity curve, and overshoot on the angle curve. We did this to ensure that the settling time thresholds scaled with perturbation size, while not changing with controller gains. The settling time landscape was again very rugged. The red dot in Fig Fb depicts the controller gains that produced the fastest settling time; this set of controller gains also caused no overshoot. Without setting force capacity limits, controller gains of 0.1560 for $\overline{K}_{p}$ and 0.6530 for $\overline{K}_{d}$ produced the fastest normalized response time ($\overline{t}_{resp}$) of 7.38 in normalized time units [(Fig Fa)](#Fig_S6).

Analysis of the relationship between force capacity limits and response time again revealed three distinct regions. The fastest response without considering force capacity limits produced a peak torque of 0.80. [Fig G](#Fig_S7) shows how response time, overshoot and controller gains changed when we varied force capacity limits from 1.2 to 0.001.

- High force capacity limits region: For $\overline{\tau}_{iso}$> 0.80, the peak torque $\overline{\tau}_{max}$ did not reach the force capacity limits, and the response time and controller gains did not change.
- Middle region: For 0.24<$\overline{\tau}_{iso}$<0.79, the force capacity limits clipped the positive region of the torque curve, and response time increased gradually with lower force capacity limits. Unlike the swing task where the controller gains did not change in the middle region, the controller gains increased gradually with lower force capacity limits.
- Low region: 0.009<$\overline{\tau}_{iso}$<0.24, the force capacity limits clipped both the positive and negative regions of the torque curve. Unlike the swing task, the response time continued to increase at the same rate as in the middle region. The controller gains initially show a shallow dip before increasing rapidly for lower force capacity limits.


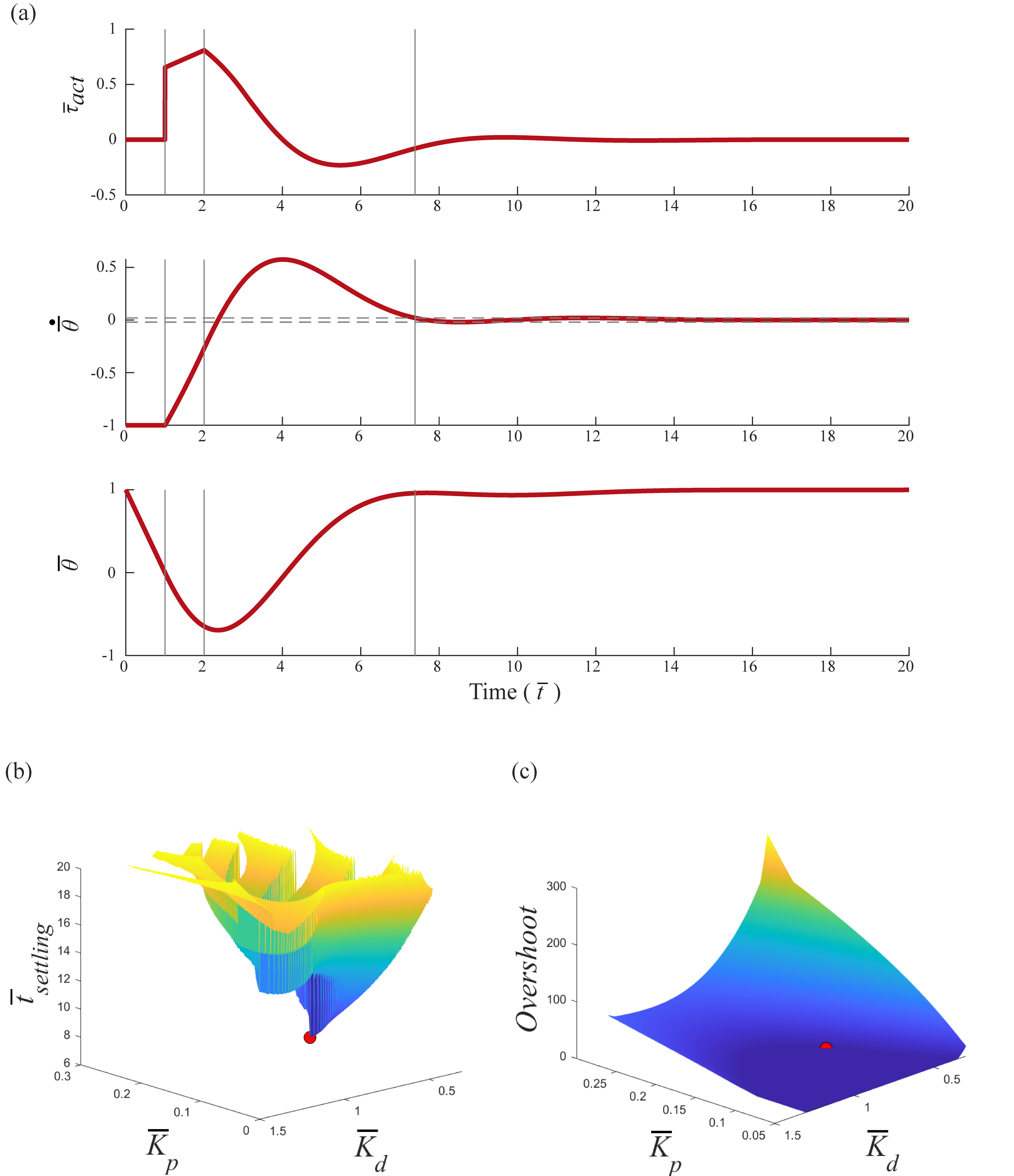


Fig F. Normalized posture task—brute force search

(a) Normalized torque ($\overline{\tau}_{act}$), angular velocity ($\dot{\overline{\theta}}$) and angle ($\overline{\theta}$) profiles in the posture task for the fastest response. The three grey vertical lines depict one time delay period, two time delay periods and the settling time. The two grey horizontal dashed lines on the angular velocity graph depict the 2% settling time thresholds. (b & c) The settling time and overshoot landscapes for the normalized posture task model determined through a brute force search for a range of controller gains. The red dot depicts the result we use for response time—the fastest settling time. The value does not change if overshoot is allowed or constrained.


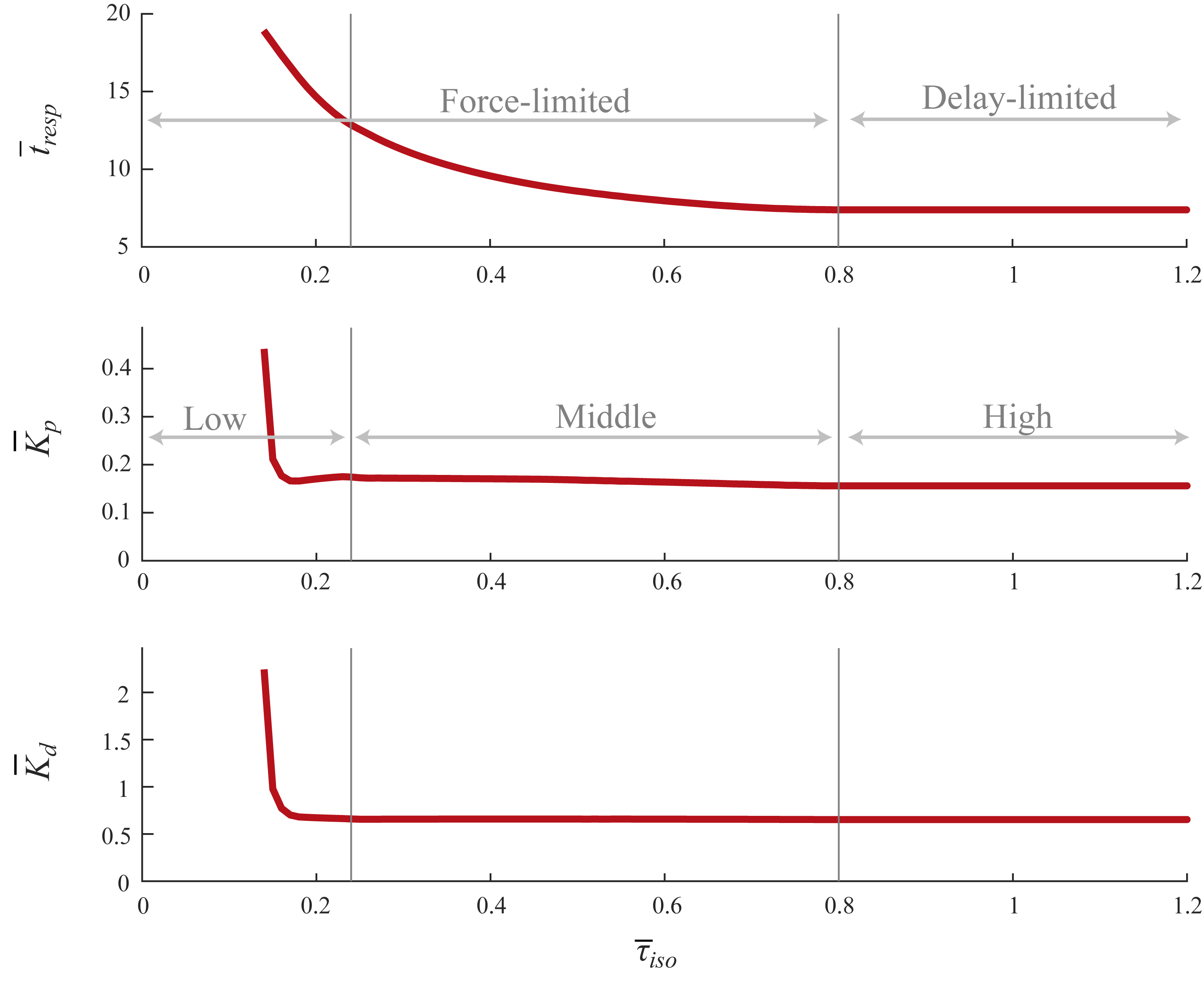


Fig G. Normalized posture task—force capacity limits vs. response time

(a) Changes in response time and overshoot when force capacity limits are lowered from 1.2 to 0.1. There are three distinct regions in the response time profile, shown divided by the grey vertical lines. (b) Changes in the optimal controller gains that produce the fastest response times without overshoot for a range of force capacity limits.

We again tried fitting various functions to the settling time curve in the middle and lower regions. We found that a power law with intercept (power2) function produced the lowest RMSE values. The posture task $\overline{t}_{resp}$ vs. $\overline{\tau}_{iso}$ relationship can be described by the following function:

$$\begin{aligned} \overline{t}_{resp}=f\left( \overline{\tau}_{iso} \right)=\left\{ \begin{matrix} 7.39 & \overline{\tau}_{iso}\geq0.80 \\ 1.61 {\overline{\tau}_{iso}}^{-1.10}+5.17 & {0.14\leq\overline{\tau}}_{iso}<0.80 \end{matrix} \right. \#S30 \end{aligned}$$

# S4. Normalized feedback model predictions vs. scaled model simulation results

Predictions for response time based on the relationship between normalized force capacity limits and response time ($\overline{t}_{resp}$ vs. $\overline{\tau}_{iso}$) from the normalized model [(Section S3)](#_S3._Normalized_feedback) compared well to results from the more detailed scaled model simulations (Section 3.2), despite their differences. The normalized models did not incorporate gravitational torques, while the scaled models considered them. Additionally, the swing task scaled simulations incorporated a steady state torque to counter gravitational torque at the target state. Feedback control response times in the scaled simulations for the swing task scaled as 199 *M*^0.21^ ms. The normalized equations [(Eqn S29)](#Eqn_S29) predicted response time from the scaled simulations with an average accuracy of -10.5% (range: -8%, -14%) [(Fig H left)](#Fig_S8). Feedback control response times in the scaled simulations for the posture task scaled as 239 *M*^0.22^ ms. The normalized equations [(Eqn S30)](#Eqn_S30) predicted response time from the scaled models with an average accuracy of 5% (range: 2%, 13%) [(Fig H right)](#Fig_S8).


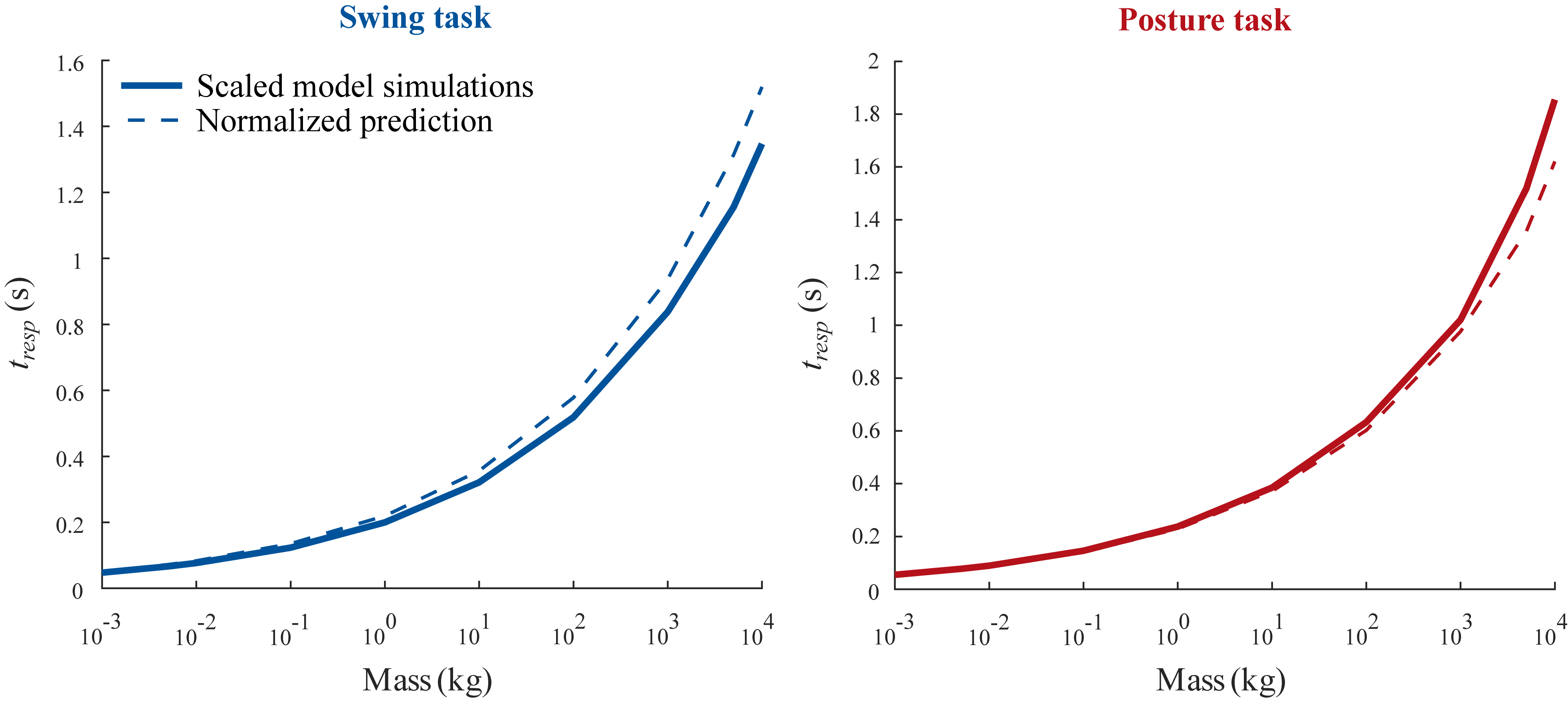


Fig H. Feedback response time—normalized model predictions vs. scaled simulation results

Normalized prediction for the swing task (left) and posture task (right) in dotted lines. Scaled simulation results in solid lines.

# S5. Components of total applied torque under feedback control

We calculated the total torque experienced by the plant as the sum of the muscle torque (generated by the feedback controller), and gravitational torque. The muscle torque composed of proportional and derivative components in the posture task, and an additional steady state component in the swing task. We capped the muscle torques at a force capacity limit, computed as the maximum isometric torque that can be produced by the relevant muscles. Applied torques exceeded force capacity limits only for animals heavier than one ton in the posture task. During an initial delay period equal to the sensorimotor delay, the controller did not apply muscle torques and the plant moved purely under gravitational torque. We considered this the time required for the animal to sense the perturbation, compute the motor commands, and transmit the signals to the muscles. After this deadtime, the controller turned on the muscle torques, computed based on time delayed state feedback. [Fig I](#Fig_S9) depicts the contribution of each component to the applied torque for a 1 kg animal in the swing task and posture task.


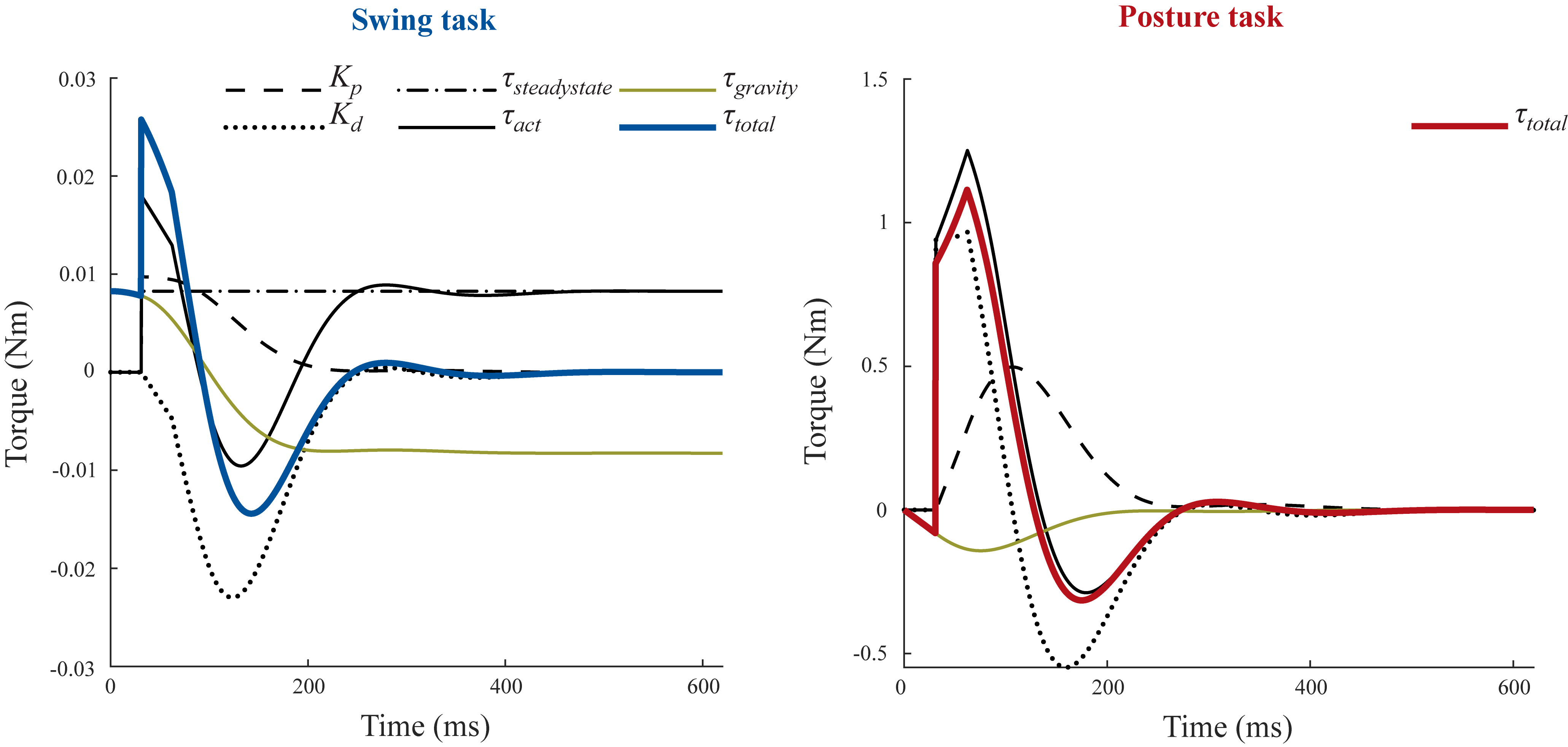


Fig I. Components of total torque

For the swing task on the left, the controller torque consisted of a proportional component ($K_{p}$—black dashed line), a derivative component ($K_{d}$—black dotted line), and a steady state component ($\tau_{steadystate}$—black dash dot line). These three components made up the muscle torque ($\tau_{act}$—solid black line). The total torque ($\tau_{total}$—thick blue line) is the sum of $\tau_{act}$ and gravitational torque ($\tau_{gravity}$—yellow green line). For the posture task on the right, the total torque is shown in red. The posture task does not have a steady state torque component. The values shown here are for a one kg animal in the swing task and posture task.

# S6. Comparing swing and posture task responses to in-vivo perturbation studies

We compared the kinematic profiles from our simulations to in-vivo perturbation studies, and found that they were qualitatively similar, despite the limitations of our computational models. In-vivo perturbation studies on animals do not elicit the fastest possible responses in order to prevent falling and injury. Our simulations model the fastest perturbation responses controlled purely through monosynaptic reflex pathways, and consider feedforward and feedback control separately. Perturbation responses from in-vivo studies are difficult to separate into purely feedforward or feedback strategies, and often involve both the reflexive and supra-spinal motor pathways. Eng et al. studied stumble corrective responses in humans, and reported that an approximately 40° swing leg repositioning response to an early swing phase trip took about 500 ms [5]. According to the swing task simulations for a 70 kg human performing a 30° swing leg repositioning, the fastest possible responses would take 172 ms under feedforward control and 487 ms under feedback control.

We also compared the posture task simulations to three studies that reported on human and cat postural responses to support-surface translations [6–8]. Horak and Nashner (1986) reported that for a 0.05 dimensionless velocity perturbation, their human subjects took about 600 ms to regain posture, and suffered a maximum lean of 3° [6]. Welch and Ting (2008) reported that for a 0.09 dimensionless velocity perturbation, their human subjects took about 1000 ms to regain posture, and suffered a maximum lean of 5° [7]. The posture task simulations predict that a 70 kg human subjected to a 0.21 dimensionless velocity perturbation would take 310 ms under feedforward control and 611 ms under feedback control to recover balance, and force them to lean to a maximum angle of about 5°. Ting and Macpherson (2004) reported that cats subjected to a 0.09 dimensionless velocity postural perturbation take about 1000 ms to regain posture, and exhibit a 5° lean [8]. The posture task simulations predict that for a 4 kg cat subjected to a 0.21 dimensionless velocity perturbation, recovering balance would take 140 ms under feedforward control and 326 ms under feedback control, and force it to lean to a maximum angle of about 3.5°. [Fig J](#Fig_S10) shows the angle vs. time profiles for different sized animals in the posture task under feedforward and feedback control. These profiles match the behavior seen in studies on posture correction in quadrupeds. The maximum lean of the posture task model (about 7° for a 0.21 dimensionless velocity perturbation) would not cause the center of mass to move outside the base of support in quadrupeds. However, we have not considered similar effects in bipeds. Bipeds would be forced to use a stepping strategy, instead of a hip or ankle strategy, if large perturbations cause the center of mass to move beyond the base of support.


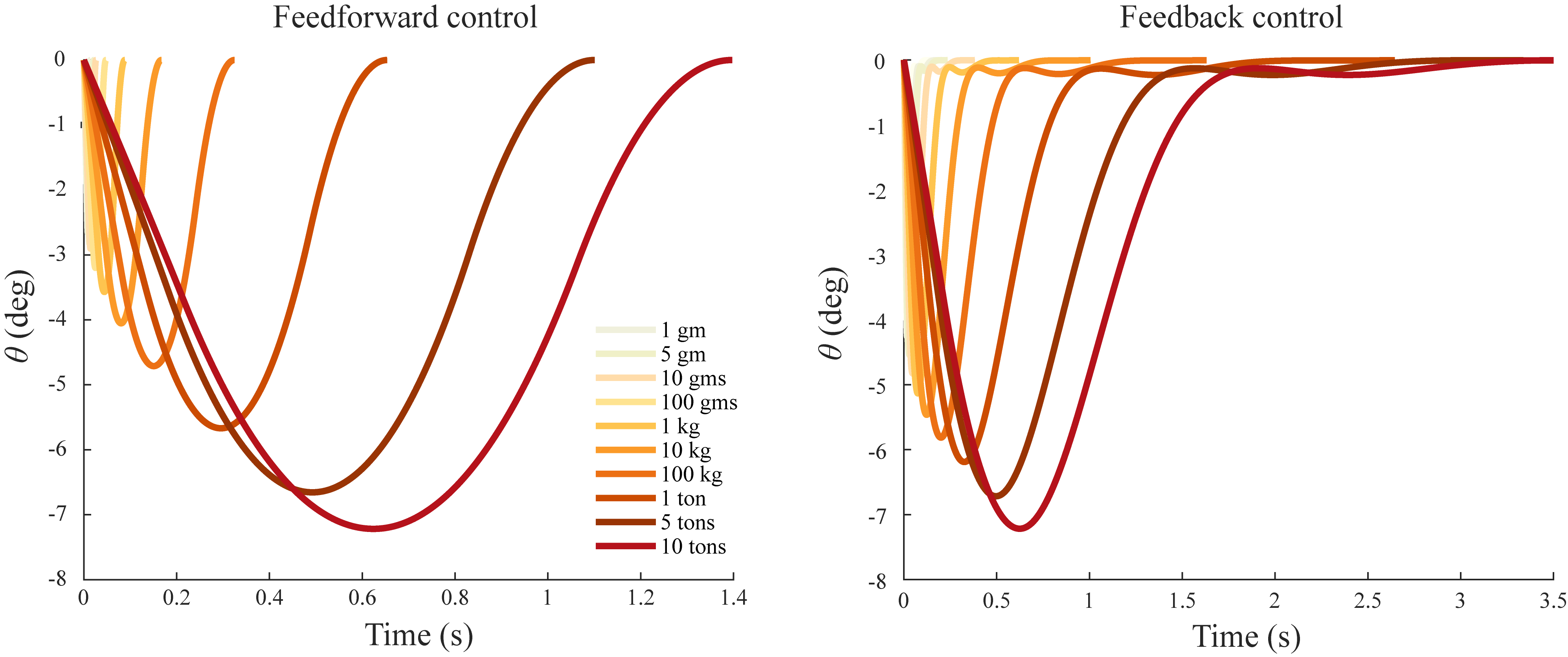


Fig J. Angle profiles for the posture task under feedforward and feedback control

Angle vs. time profiles from the posture task simulations for animal sizes ranging from 1 gram to 10 tons under feedforward control (left) and feedback control (right), for a 0.21 dimensionless velocity perturbation.

# References

1. More HL, Hutchinson JR, Collins DF, Weber DJ, Aung SKH, Donelan JM. Scaling of sensorimotor control in terrestrial mammals. Proc R Soc B Biol Sci. 2010;277: 3563–3568.

2. Åström KJ, Murray RM. Feedback systems: an introduction for scientists and engineers [Internet]. Princeton University Press. Princeton, New Jersey: Princeton University Press; 2008. Available: http://www.cds.caltech.edu/∼murray/amwiki

3. Ruina A, Pratap R. Introduction to Statics and Dynamics. Oxford University Press, Inc. (Preprint); 2015.

4. Nise NS. Control Systems Engineering. 6th ed. John Wiley & Sons Inc.; 2011.

5. Eng JJ, Winter DA, Patla AE. Strategies for recovery from a trip in early and late swing during human walking. Exp Brain Res. 1994;102: 339–349. doi:10.1007/BF00227520

6. Horak FB, Nashner LM. Central programming of postural movements: adaptation to altered support-surface configurations. J Neurophysiol. 1986;55: 1369–1381. doi:10.1152/jn.1986.55.6.1369

7. Welch TDJ, Ting LH. A feedback model reproduces muscle activity during human postural responses to support-surface translations. J Neurophysiol. 2008; doi:10.1152/jn.01110.2007

8. Ting LH, Macpherson JM. Ratio of Shear to Load Ground-Reaction Force May Underlie the Directional Tuning of the Automatic Postural Response to Rotation and Translation. J Neurophysiol. 2004;92: 808–823. doi:10.1152/jn.00773.2003
