# Supplementary material for: Effects of sensorimotor delays and muscle force capacity limits on the performance of feedforward and feedback control in animals of different sizes
Source: PLoS Comput Biol. 2026 Apr 21;22(4):e1012502. doi: 10.1371/journal.pcbi.1012502 (PMC13152214; doi:10.1371/journal.pcbi.1012502)
Supplement: Attachment — Submitted filename: Scaling of feedback control-Response to reviewers.pdf [file pcbi.1012502.s002.pdf]

Response to Reviewers

PCOMPBIOL-D-24-01586

Effects of sensorimotor delays and muscle force capacity limits on the performance of feedback control in animals of different sizes

Sayed Naseel Mohamed Thangal, Heather L. More, C. David Remy and J. Maxwell Donelan

Reviewer #1: Sensorimotor delays and muscle force capacity scaling for control of movement

The authors provide two simple models for testing the relative efficacy of feedback versus feedforward control of (1) a (fore)limb swing perturbation recovery task versus (2) a body CM support (hind)limb recovery task to evaluate how body size (mass) affects these two differing control strategies. In doing so, they build on earlier experimental and modeling work that evaluated how sensorimotor delays and inertial effects scale with animal size. Their modeling here shows that small animals may well benefit from feedforward control of perturbations for recovery from fast movements; whereas, both small and larger animals face challenges due to sensorimotor delays in responding to fast perturbation challenges under feedback control. The introduction does a nice job of providing a broader context for their modeling study. However, while I generally enjoyed reading their paper, I was disappointed by the sloppy organization of the methods and results, with figures out of sequence and several terms and parameters that were not defined or explained clearly. This made for a confusing read at times. I am not a controls engineer, so several detailed aspects of control theory and elements of their model are not ones that I can evaluate critically. But there are other areas (from the biological/biomechanics side) in which I am familiar, and certain specific aspects of their models need to be better justified and explained. With improved organization and clear explanation of terms, and definition of parameters, this will make a valuable paper to build on the lead (corresponding) author's work. In general, the paper could benefit from being more concise (this also applies to the discussion, which is rather long and overly speculative in places).

Thank you for the careful and insightful read of our manuscript. As detailed below, we have taken care to improve on its organization, clarity, justification of modeling choices, and conciseness. We feel it has improved greatly with your feedback, and we hope you find the same.

My specific comments follow:

1. l. 56-57 – need citations to Daley & colleagues' relevant papers here, presumably [7-11],

or identify them more specifically through l. 64. Leaving the [7-11] citation at l. 65 makes it unclear which of the preceding statements related to the citations [7-11].

In response to reviewer 2's comment 2 on feedforward control, we have significantly changed the second paragraph of the Introduction. We now only make a single statement about Daley & colleagues' studies and have cited three relevant articles at the end of the sentence. It now reads: *"Running guinea fowl encountering obstacles maintain rhythmic feedforward control of proximal leg joints, while distal joints respond preflexively [1–3]."*

2. l. 63 & l. 68: it might be helpful to define what you mean by 'preflexively' or 'preflexes' for a general audience.

Thank you for the suggestion. This sentence now reads: *"The musculoskeletal system can also react passively to stabilize movement against perturbations. Known as preflexes, these intrinsic responses act instantaneously and are not reflex-pathway mediated, although they are modulated by the active state of muscles and joint positions during movement [4]."*

3. l. 106: I appreciate the citation to [29], but this (though the first) was a limited study on the scaling of stride frequency vs body size (based on relatively few species), which did not focus on the time duration for correcting a disturbance (stride or step period) – which is the point the authors wish to make here. I think making the link between stride frequency, step/stride duration, and response time to disturbances for control of movement is needed vs this brief statement and citation to [29]. Perhaps one or more other papers should be cited? Also, Heglund & Taylor (JEB, 1988) provides stronger evidence for the scaling of stride frequency than the Science [29] paper.

We have now added 2 additional citations and have changed the original #29 citation to the Heglund and Taylor 1988 JEB paper. This sentence now reads: *"For example, stride frequency decreases with increasing body size, as shown by Heglund and Taylor (1988) across a broad range of terrestrial mammals [5], leading to longer stride and swing durations [6,7]. Since swing duration determines the time window to reposition a limb after a trip, larger animals have more time to complete such corrective responses during locomotion."*

4. Fig. 1 (& Methods) – use of a bang-bang controller for feedforward activation/control should be explained and refs cited.

In response to your feedback and reviewer 2's comments, we now explain bang-bang control and provide relevant citations in the Introduction, Figure 1 caption and in the Methods sections.

The modified second paragraph of the Introduction now reads: *"The fastest neurally mediated control strategy to reject a perturbation is a type of feedforward control called "bang-bang*

control" [8,9] . It is an open-loop, pre-planned motor command computed assuming perfect knowledge of the system and perturbation [10–12]. As such, it represents the theoretical upper limit of response speed achievable by a control strategy, constrained only by initial sensorimotor delay, actuator force capacity, and system dynamics."

The caption of Figure 1 reads: *"The feedforward system (bottom) uses a bang-bang controller to generate torque—a control strategy that switches instantaneously between maximum muscle flexion and extension forces [8–12]. It is a time-optimal, open-loop strategy that precomputes the required response based on known perturbation characteristics, serving as an upper-bound benchmark for the fastest possible perturbation response."*

The Methods section reads: *"Under feedforward control, the plant dynamics are identical but the actual torque (τ_{act}) represents the torque produced by a bang-bang controller—a control strategy that switches instantaneously between our estimates of the maximum isometric flexion and extension torques [9,11,12]."*

5. I am most concerned by the non-physiological square-wave input torque control of this approach (1B). Authors should explain and justify this, beyond presenting the conditions at l. 197. (1A) The rapid rise in input torque for feedback control also seems non-physiological. Can the authors explain/justify this?

We agree that the instantaneous torques applied by the controllers are not strictly physiological. This was a choice to keep our models simple and tractable. More complex muscle models would outstrip the realism of the rest of our modeling that employs rigid segments, hinge joints, no soft tissue, no actual nerve fibers, etc. And even if we were to attempt to include more realistic muscle models, there is a lack of data to reasonably scale muscle model parameters across the size range of terrestrial mammals. Instead, our sensorimotor time delay parameter includes two components—electromechanical delay and force generation delay—which roughly approximate how muscle takes time to generate force [7]. The electromechanical delay approximates the lag in force generation caused by muscle activation-deactivation dynamics. The force generation delay approximates the time required for muscle fibers to reach peak twitch force. We now explain these delays in the Methods section, and discuss this limitation in the second paragraph of the Discussion. The relevant sentences read:

In the Methods section 2.1.3: *"As we use simple controllers, both the feedforward bang-bang controller (Eqn 5) and the feedback PD controller (Eqn 4) apply instantaneous torques after the initial deadtime. We did not model Hill type muscles due, in part, to a lack of data to reasonably parametrize such models across the size range of terrestrial mammals. Instead, our sensorimotor time delay parameter includes two components—electromechanical delay and force generation delay—which roughly approximate how muscle takes time to generate force and how it scales across body size [7]. The electromechanical delay approximates the lag in force generation*

caused by muscle activation-deactivation dynamics. The force generation delay approximates the time required for muscle fibers to reach peak twitch force."

In the Discussion para 2: *"We have approximated gradual changes in muscle force with fixed electromechanical and force generations delays resulting in instantaneous changes in our modeled muscle torques that are non-physiological. While this is not a limitation of some dynamic models of muscle behaviour (e.g. Hill type), we chose not to incorporate them for two reasons: it keeps our models simple, and thus tractable, and because there is a lack of data to accurately scale muscle-tendon architecture across the size range of terrestrial mammals."*

6. l. 182: " des" is not defined – an equation at l. 187 is given but no description of what the parameter represents [it is defined on p. 17 at l. 294!]

Thank you for pointing this out. Our wording made it easy to miss definitions. We have reworded to improve clarity and also italicized the definition of terms. The sentence now reads "τ_{des} represents the *desired torque*".

7. p. 13: " SM" is also not defined [it is eventually defined on p. 17 at l. 300!]

We have italicized term definitions to improve readability.

8. l. 235-236: Why are data for the scaling of the triceps muscle from Alexander et al 1988 used to parameterize shoulder muscles? Triceps acts mainly at the elbow in mammals, including humans. It is not a muscle that will swing the forelimb forward at the shoulder or support body weight related forces during stance (posture task). Deltoid, pectoralis supraspinatus would be more anatomically and functionally relevant.

This is a good point. While one of the three heads of the triceps—the triceps brachii caput longum—does connect to the scapula and flex the shoulder joint [13], we acknowledge that the triceps is not the main muscle moving the shoulder joint. We used scaling data for the triceps from Alexander et. al. (1981) to parameterize the shoulder flexors and extensors, as it is the only reference we could find that provided the parameters necessary to estimate muscle torque (muscle mass, muscle length and muscle moment arm) [14]. Bishop et. al. more recently published scaling data for muscles, grouping all muscles above the elbow together as proximal muscles [15]. Their scaling data for proximal muscle mass and length are similar to the values we use, but they do not provide values for muscle moment arms. We added to the Method section to elaborate on our decision making. It reads:

"We estimated the maximum force capacity of the shoulder muscles (τ_{iso}) using muscle mass, length and moment arm data for the triceps published by Alexander et al. (1981) [14]. While one of the three heads of the triceps—the triceps brachii caput longum—does connect to the scapula and flex the shoulder joint [13] , it is not this joint's main muscle. We nevertheless used

Alexander's scaling data for the triceps to parameterize the shoulder flexors and extensors, as it is the only literature reference that provides the parameters necessary to estimate muscle torque. Bishop et. al. found similar scaling relationships as those we used here for fore limb proximal muscle mass and length [15] . However, they did not provide values for muscle moment arms, so we elected to use Alexander's triceps values."

9. L, 248-249: I was initially confused by the authors stating that the posture perturbation control task is achieved by the plantarflexors. This is because no clear explanation of the two tasks is provided at the outset of the paper. In defining and modeling these two tasks (swing vs posture) the authors need to make clear that the swing task concerns a perturbation to the swing of the forelimb – controlled at the shoulder versus a posture task that involves control of a perturbation causing an angle change in the supporting hindlimb to control CM motion; making it clear that two different sets of muscles (and limbs) are involved in these two tasks.

Thank you for this feedback. We read through from the start of the manuscript trying to determine the optimal location to clarify the issue that led to your confusion (and thus the likely confusion of future readers). We found that the best place for this clarification was at the beginning of each section that describes the tasks in detail. These subsections are labeled as "Plant dynamics" and now read:

*"**Plant dynamics.** The swing task represents a forelimb trip during early swing, requiring the animal to use its shoulder muscles to reposition its leg to prevent a fall. This is similar to the stumble corrective response in animals [16,17] and the elevating strategy in humans [18]."*

And…

*"**Plant dynamics.** The posture task represents an animal recovering its posture after a forward push to the body, under the control of the ankle joint plantarflexors."*

We also added clarifying text to the last paragraph of the Introduction. It now reads *"Consequently, we quantified response times for two perturbation response tasks: a swing leg repositioning task under the control of shoulder muscles (swing task), and a posture recovery task under the control of ankle joint muscles (posture task)."*

10. This is clearly stated at l. 499-500 but needs to be made clear in Methods when first defining these tasks. But again, re: l. 499-500, the triceps do not control swing motion at the shoulder. I know of no evidence for this in any mammal.

We have updated the last paragraph of the Introduction and the Plant dynamics paragraphs in the Methods section to address your comment. Please refer to our response above to comment 8

regarding using triceps to approximate the actual muscles used to actuate the shoulder.

11. Fig. 2 (p. 26!) is not referenced or discussed prior to Fig. 3 at l. 170. Much of the explanation of Fig. 2 in the legend is not clearly explained or justified as a result.

We refer to Fig 3 before Fig 2 due to organizational choices we made to keep the paper concise and avoid repetition. In the Methods section, we describe our more elaborate scaled models first and the normalized model second, to avoid having to describe equations in both expanded and simplified forms and repeat term definitions. But in the Results, we describe the predictions from the normalized model first and then describe how the scaled model results agreed with the predictions. To clarify our approach to readers, we have added a description of this organizational choice in the Methods section:*"To improve clarity and avoid repetition, we have reversed the order of subsections within the Methods and Results sections. In this section, we first describe our more elaborate scaled models (2.1) and then list the simplifications made to develop the normalized feedback model (2.2). In the subsequent Results section, we first describe predictions from the normalized model (3.1), and then validate those predictions on our scaled models (3.2)."*

Thank you for pointing out this issue with the Fig 2 caption. In section 3.1, we have moved significant parts of the analysis out of the caption for Fig 2, and back to the text.

12. Fig. 3(B) p. 28: please explain justification for the instantaneous ramp in torque at 31 ms.

Please refer to our response to comment 5 above. Here are a few additional details:

In Fig 3B between 0 and 31 ms, due to sensorimotor delays in the sensory feedback pathways, the controller does not know that the swing limb has been perturbed. Thus, the torque applied between 0 and 31 ms is purely gravitational. At 31 ms, the time delayed sensory feedback reaches the PD controller, which triggers a jump in torque due to the large error between the time delayed limb angle $\theta(t - t_{SM})$ and reference angle θ_r. This corresponds to the proportional term ($K_p[\theta_r - \theta(t - t_{SM})]$) in Eqn 8. In supplementary section S5, we provide a breakdown of the components of the applied torque.

13. l. 396: Also, why is Fig. 3 titled this way: "Fig 3. Scaled model—swing task responses under feedback control"? Feedforward control is also shown in comparison to feedback control.

Thank you for catching our mistake. This title now reads: "Fig 3. Scaled model—swing task control".

14. l. 443: This also applies to Fig. 5 "Fig 5. Scaled model—posture task responses under feedback control", which also shows a comparison of feedback vs feedforward control in panel C. Hence, figure titles for figs. 3 & 5 are inconsistent with what is shown.

As with the above comment, this title now reads: "Fig 5. Scaled model—posture task control"

15. l. 410: How was swing duration at maximum sprint speed determined? [19,27] are cited but referencing these studies does not explain how EITHER maximum sprint speed vs animal body mass or swing duration were calculated. [19,27] report sensorimotor and inertial delays – not maximum sprint speeds or swing durations. Maximum speeds of terrestrial animals are notoriously noisy and difficult or unreliable to determine.

We agree that this was originally unclear. However, rather than detail here how this scaling relationship is determined, we have clarified that this relationship is derived in prior work. This content now reads: *"After More & Donelan and Thangal & Donelan, we estimated the shortest time available to complete a perturbation response for animals of different sizes as the swing duration at maximum sprint speed [7,19]. Table 1 provides the coefficients for this scaling relationship—we refer readers to the original papers for its derivation."*

16. l. 435-437 "The ratio of feedback response times to feedforward response times ranged from four in smaller animals to one and a half in larger animals (Fig 6b)." This statement doesn't match well what Fig. 6b shows, which by my eye ranges from <4 (~3.8?) to ~1.3 not 1.5.

To increase clarity and precision, we have put in exact values. This now reads: *"The ratio of feedback response times to feedforward response times ranged from 3.8 in smaller animals to 1.3 in larger animals (Fig 6b)."*

17. l. 454-455: This statement is presumably in reference to Fig. 6C, but this is not made clear. Explicit reference to the figures would help make the presentation of your results clearer.

We have made this change.

18. l. 505: "we used … a single pair of opposing muscles to actuate them [26,44]." I don't follow this statement. No antagonistic pair of muscles is presented for controlling either the shoulder in the swing task or the ankle (plantarflexors) of the posture task. Only one set for each task.

The "muscle" torque, τ_{act}, can exert both extensor or flexor torques for both the swing and posture tasks, representing the actions of antagonist extensor and flexor muscles. To make this clearer, we have added the following sentence to the Plant Dynamics subsection for both tasks: *"This torque can be negative-valued or positive-valued where the sign indicates whether the extensor muscle or the antagonist flexor muscle is active."*

19. l. 514-515: provide (textbook) references for these complex feedback controllers?

We have added references that describe Model Predictive Control and Smith Predictors [20,21].

20. Paragraph from l. 519-534 is overly speculative, as the experimental techniques suggested are close to being unrealistic: "Experiments of this nature would be challenging to carry out in animals spanning the full size range of terrestrial mammals." An understatement if there ever was one!

We have modified the sentence to describe studies suggested by reviewer 2 in comment 5, on how perturbation experiments in different species can shed light on how neural controllers integrate feedforward and feedback control. It is perhaps worth noting that our lab has successfully measured conduction velocities in animals ranging from shrews to elephants. And that the cited techniques in this paragraph have all been done in at least one species of terrestrial mammals.

21. l. 564: I think I know what is meant by 'sensory dead zones', but some definition or clearer statement of what is meant would be helpful.

This concept was not central to our point, so we have removed "sensory dead zones" from that list.

22. l. 584: define 'damping ratio'

We rewrote this content to eliminate the need for the term "damping ratio" so a definition is no longer needed. It now reads: *"Garcia et al. (2000) showed that the limbs of small animals are overdamped while the limbs of larger animals are underdamped [22]. Overdamping will help prevent overshoot or oscillations when repositioning the body making small animals less sensitive to modeling errors in feedforward control."*

23. l. 600: "on more computational[ly intensive] control strategies"

We have made this excellent suggested change. Thank you.

24. l. 752-755: ref 56 is quite odd, with information irrelevant to the paper cited.

We respectfully disagree. This paper describes how one can experimentally manipulate control system time delay in behaving animals. According to Google Scholar, it has been cited 48 times, including 8 times since 2021. We have elected to keep it in.

Reviewer #2: In this paper, Thangal et al carry out a computational modeling study investigating the role that sensory delay and actuator force saturation play in limiting the response times of a feedback controller. This study is carried out on two simulated plant models: a swinging limb in gravity and a postural balance task. They compare feedback (FB) control performance to a feedforward (FF) controller and conclude. Care is taken to dimensionally scale the system and control across an extremely wide size range.

1. First, let me focus on the feedback control conclusions. I think there is value in their feedback control analysis particularly for the scaled models. The main idea in the paper is simple: feedback is generally delay limited, and not actuator limited, in producing the "fastest" response to a perturbation. This is perhaps obvious at some scales, but it is not obvious how this will scale across a wide range of animal sizes nor was it obvious to me if or when one might see a crossover between force- vs. delay limitations. After reading the paper, this made sense but it was interesting and thoughtful. I will say that I don't "believe" the exact cross over point because I think that one thing the model doesn't take into account is that damping becomes dominant at lower amplitudes, for example, and smaller animals are often somewhat more sprawled. But the general trends and scaling relationships are nevertheless interesting and one can't expect for a simplified model that scales from mice to elephants to get it all right for everything. That's not the intent of the modeling. In short, this part seems a solid result and is the main strength of the manuscript, building nicely on lab's prior work on scaling of feedback delay.

Thank you for this supportive feedback.

2. However, the FF control feels like a straw man. Normally, one thinks of a FF motor program as a set of motor commands that are driven e.g. by a CPG and not subject to sensory feedback. Of course most researchers including the authors recognize that this is an over simplification as CPGs are generally modulated in relation to ongoing sensory feedback, but the idea is that the time scales of that feedback are much longer than the strong initial responses that the system can provide to perturbations. This is beautifully illustrated in the paper they cite by Jindrich and Full showing that peak lateral leg forces due to an impulsive perturbation to a cockroach occur far faster than could be expected from neural reflexes, or the work by Daley's group on perturbations to running bipedal birds, as also cited. However, these perturbation responses are caused by musculoskeletal interactions with the environment, e.g. passive viscoelastic responses of muscles—critically

those responses can be tuned based on ongoing motor programs and therefore create a "preflexive" response. So,I had expected when reading the abstract, e.g. to see two muscles fighting each other, and increasing tonic antagonistic activation and showing using a muscle model how the muscular responses gets closer to maximum with a perturbation the higher the tonic antagonistic activation. Instead, the authors proposed an *EXACT* bang-bang controller that moves you from a perturbed position to the home position. FF control can't do this. It requires feedback to exactly cancel out a perturbation in the way proposed. This is especially true for the unstable model. In other words, creating a pre-planned motor program that must be exactly tuned to the perturbation is not "FF control". Also, it is well known that the best way to do this in controls to achieve a minimum time response is typically bang bang at the actuator limits so it is a foregone conclusion that it will always hit the maximum force.

We appreciate the reviewer's concern that our implementation of feedforward control as an exact, time-optimal bang-bang controller may appear as an unrealistic "straw man" when compared to biological motor control strategies. Indeed, biological feedforward control is not limited to the precise, perturbation-specific solutions that we model but can also leverage preflexive musculoskeletal dynamics, co-contraction, and robust approximations. However, our intent was not to model the full richness of biological feedforward mechanisms, but rather to represent the idealized performance limit of open-loop, non-feedback control in the face of perturbations. In control theory, bang-bang control represents the time-optimal solution for systems with input constraints and full knowledge of perturbation characteristics [8–12] . By using this formulation, we establish an upper bound on what any purely feedforward controller—irrespective of biological complexity—could achieve in terms of response speed. This abstraction serves as a useful counterpoint to highlight the scaling limitations of feedback control under delay constraints. Furthermore, the notion of feedforward as a pre-computed, model-based action without reliance on output feedback is consistent with formal definitions in control engineering [23,24] and captures essential aspects of ballistic motor programs and anticipatory postural adjustments observed in biological systems [25,26] . We agree that incorporating musculoskeletal dynamics would enrich biological realism, but such additions would reduce the system's time-optimal performance relative to our bang-bang benchmark—not enhance it. Thus, while our feedforward implementation is intentionally idealized, it is not a straw man; rather, it is a theoretically justified feedforward exemplar that serves to frame the limitations of feedback-dominated responses across scales.

We acknowledge that our original writing did not make the above points clear, especially considering your criticisms. To address our shortcoming, we have revised the Title and the Abstract, as well as extensively revising the Introduction. Below we describe these changes:

Title
We have removed the term "feedforward" from the title. The title now reads: *"Effects of sensorimotor delays and muscle force capacity limits on the performance of feedback control in animals of different sizes."*

Abstract
The Abstract now primarily focuses on feedback control. We introduce feedforward control towards the end and describe it as "the fastest feedforward control strategy" to denote it as one of many. The final sentence adds descriptors to some of the various types of feedforward control so that it is clearer to the reader that we don't just mean CPGs. It reads: *"Thus, feedforward control strategies—including anticipatory adjustments, ballistic motor programs, and exploitation of intrinsic musculoskeletal dynamics—may be essential for reacting quickly to sudden and large perturbations in animals of all sizes."*

Introduction
We introduce biological control strategies in the second paragraph of the Introduction. Originally, this paragraph was biased towards examples of feedforward control, and those examples were biased towards evidence of preflexes. We have extensively revised this paragraph to include more biological examples of feedforward control—including ones more like our modeled open-loop pre-planned feedforward control—and more examples of biological feedback control. This paragraph is too long to quote here.

The third paragraph was originally about how time delays and actuator force capacity limit the performance of feedback and feedforward control systems. We have now divided that paragraph into two paragraphs and added clearer content about how we model feedforward control at the end of the first of the two paragraphs. It attempts to convey that our feedforward control is just one type of feedforward control, and most importantly, why we have chosen to study this control strategy. It reads: *"The fastest nerually mediated control strategy to reject a perturbation is a type of feedforward control called "bang-bang control" [8,9] . It is as an open-loop, pre-planned motor command computed assuming perfect knowledge of the system and perturbation [10–12]. As such, it represents the theoretical upper limit of response speed achievable by a control strategy, constrained only by initial sensorimotor delay, actuator force capacity, and system dynamics."*

We also modified the last paragraph of the Introduction where we describe our objective. It now reads as follows, with the bolded content designed to directly address your concerns: *"Here our objective was to understand the performance of feedforward and feedback control strategies in animals of different sizes when constrained by sensorimotor delays and muscle force capacity limits. To accomplish this, we developed simple feedforward and feedback control systems and*

scaled them to represent the size range of terrestrial mammals. ***Importantly, our feedforward controller was designed as a time-optimal, open-loop benchmark—an idealized representation of the fastest possible response achievable, given perfect knowledge of the perturbation and system dynamics. This abstraction serves to define an upper bound on feedforward performance, distinct from passive feedforward strategies that exploit intrinsic musculoskeletal dynamics.*** *The consequences of time delays and actuator force capacity are most evident when a system is required to respond as fast as possible, because fast responses require using the maximum possible muscle forces while ensuring that the response remains accurate and stable."*

Finally, we added this content to the Figure 1 caption where the two control strategies are diagramed: *"The feedforward system (bottom) uses a bang-bang controller to generate torque—a control strategy that switches instantaneously between maximum muscle flexion and extension forces* [8–12]. *It is a time-optimal, open-loop strategy that precomputes the required response based on known perturbation characteristics, serving as an upper-bound benchmark for the fastest possible perturbation response."*

3. So, in sum, the FB control modeling is interesting and informative, but the lack of muscle dynamics and other passive mechanisms in the model (which are partially described in the intro) that integrate with a more appropriate form of FF control is a major weakness of the paper. The FF "control" described herein is more like a pre-programmed ballistic movement and so far as I can tell has nothing to do with rejecting perturbations. Ideally the FB controller would also include a muscle model.

We appreciate your perspective on these issues. In our response to the previous comment, we discuss how our pre-programmed feedforward control serves as an upper bound for all perturbation rejection control strategies. So, in this response we will focus on addressing the need for modeling muscle and other passive mechanisms.

We do already model aspects of muscle dynamics. The sensorimotor delay from More and Donelan (2018) is the sum of several component delays including electromechanical delay and force generation delay [7]. Electromechanical delay is the time for the action potential to be conducted along muscle fibers and for molecular mechanisms involved in cross-bridge formation to be activated, and force generation delay is the time for the muscle to develop peak twitch force. Your comment brought to mind the quote that "a model should be as simple as possible, but not too simple". We feel that our very simple model of muscle dynamics is appropriate for the questions we are trying to answer here, and more bio-fidelity in our muscle modeling would be a mismatch for the complexity at which we model other aspects of the neuromechanical system. But the quoted heuristic does not provide actual guidance about what is too simple, and different scientists can reasonably disagree as to where this lies.

We acknowledge that this model of muscle dynamics is far simpler than the more conventionally used Hill-type models. For example, it does not have a series elasticity, nor does it have short-range stiffness [27], both of which would be useful for studying preflexive responses to perturbations. While it would be both interesting and important to study the scaling of preflexive responses, that is not one of the objectives of the present paper. Furthermore, it's not clear to us that it would be possible to do at present as we do not know the scaling relationships for several muscle model parameters across the size range of terrestrial mammals. Even if these relationships existed, we suspect that attempting to include such scaled muscle models would obscure the insights into how sensorimotor delays and force capacity limits interact to affect response times in this paper.

4. I see a few ways of going — cutting FF altogether and having a much short communication, or dramatically improving the FF model. My idea above may be terrible so I'm not advocating a particular solution, but rather the model should capture the essence of preflexive perturbation responses.

We hope that our response to Comment 2 has convinced you that our feedforward modeling is valuable in meeting the objectives of our manuscript. We also hope that our response to Comment 3 has convinced you that the scaling of preflexive perturbation responses, while both interesting and important, is beyond the scope of the present manuscript.

5. Also, the FF/FB hypothesis has a rich history, much of which the authors cited. A couple other papers of interest that nicely describe FF/FB axis:
Revzen, S., Koditschek, D.E., Full, R.J. (2009). Towards Testable Neuromechanical Control Architectures for Running. In: Sternad, D. (eds) Progress in Motor Control. Advances in Experimental Medicine and Biology, vol 629. Springer, Boston, MA.
https://doi.org/10.1007/978-0-387-77064-2_3
Holmes, Philip, et al. "The dynamics of legged locomotion: Models, analyses, and challenges." SIAM review 48.2 (2006): 207-304.

Thank you for these suggested references. We have now added them.

6. Minor: the %OS graph being constant seems unnecessary.

Thank you for this suggestion. We have removed it.

7. There are a number of editing issues, but one that was particularly confusing was on p29 "We estimated time available to complete the pertubation response as the time…" do you mean "as a fraction of the time?

Thank you for pointing out this potential source of confusion. Our intent was to define the available response time explicitly as the time it would take to fall under gravity from a height equal to leg length. To clarify this, we have revised the sentence to read: *"We estimated the time available to complete the perturbation response by defining it as the time required to fall under gravity to the ground from a height equal to the leg length for each animal size…"* This wording avoids the ambiguity of "as" and makes our definition clearer.

Have the authors made all data and (if applicable) computational code underlying the findings in their manuscript fully available?

We have shared the computational code underlying our findings through the following Github repository:
https://github.com/sayednaseel/GITHUB-ScalingOfFeedbackControl

Figure resubmission:
While revising your submission, please upload your figure files to the Preflight Analysis and Conversion Engine (PACE) digital diagnostic tool, https:// pacev2.apexcovantage.com/. PACE helps ensure that figures meet PLOS requirements. To use PACE, you must first register as a user. Registration is free. Then, login and navigate to the UPLOAD tab, where you will find detailed instructions on how to use the tool. If you encounter any issues or have any questions when using PACE, please email PLOS at figures@plos.org. Please note that Supporting Information files do not need this step. If there are other versions of figure files still present in your submission file inventory at resubmission, please replace them with the PACE-processed versions.

We have used the NAAS tool provided by PLOS to review our figures, and confirm that all our figures passed PLOS specifications.

References

1. Daley MA, Biewener AA. Running over rough terrain reveals limb control for intrinsic stability. Proceedings of the National Academy of Sciences. 2006;103: 15681–15686. doi:10.1073/pnas.0601473103
2. Nishikawa K, Biewener AA, Aerts P, Ahn AN, Chiel HJ, Daley MA, et al. Neuromechanics: An integrative approach for understanding motor control. Integr Comp Biol. 2007;47: 16–54. doi:10.1093/icb/icm024
3. Daley MA, Usherwood JR, Felix G, Biewener AA. Running over rough terrain: guinea fowl maintain dynamic stability despite a large unexpected change in substrate height. Journal of experimental biology. 2006;209: 171–187.
4. Brown I, Loeb G. A reductionist approach to creating and using neuromusculoskeletal models. Biomechanics and neural control of posture and movement. 2000; 148–163. doi:10.1007/978-1-4612-2104-3_10

5. Heglund NC, Taylor CR. Speed, stride frequency and energy cost per stride: how do they change with body size and gait? J Exp Biol. 1988;138: 301–318.
6. Alexander RM, Jayes AS. A dynamic similarity hypothesis for the gaits of quadrupedal mammals. J Zool. 1983;201: 135–152.
7. More HL, Donelan JM. Scaling of sensorimotor delays in terrestrial mammals. Proceedings of the Royal Society B: Biological Sciences. 2018;285: 20180613. Available: http://dx.doi.org/10.1098/rspb.2018.0613
8. Rao VG, Bernstein DS. Naive control of the double integrator. IEEE Control Systems Magazine. 2001;21: 86–97. doi:10.1109/37.954521
9. Nelson WL. Physical principles for economies of skilled movements. Biol Cybern. 1983;46: 135–147.
10. Libby T, Johnson AM, Chang-Siu E, Full RJ, Koditschek DE. Comparative Design, Scaling, and Control of Appendages for Inertial Reorientation. IEEE Transactions on Robotics. 2016;PP. doi:10.1109/TRO.2016.2597316
11. Tsianos GA, Rustin C, Loeb GE. Mammalian muscle model for predicting force and energetics during physiological behaviors. IEEE Transactions on Neural Systems and Rehabilitation Engineering. 2012;20: 117–133. doi:10.1109/TNSRE.2011.2162851
12. Wong JD, Bobbert MF, Van Soest AJ, Gribble PL, Kistemaker DA. Optimizing the distribution of leg muscles for vertical jumping. PLoS One. 2016;11. doi:10.1371/journal.pone.0150019
13. Böhmer C, Theil J-C, Fabre A-C, Herrel A. Atlas of Terrestrial Mammal Limbs. Boca Raton: CRC Press; 2020.
14. Alexander RM, Jayes AS, Maloiy GMO, Wathuta EM. Allometry of the leg muscles of mammals. J Zool. 1981;194: 539–552. doi:10.1111/j.1469-7998.1981.tb04600.x
15. Bishop PJ, Wright MA, Pierce SE. Whole-limb scaling of muscle mass and force-generating capacity in amniotes. PeerJ. 2021;9: e12574.
16. Rossignol S, Dubuc R, Gossard J-PP. Dynamic Sensorimotor Interactions in Locomotion. Physiol Rev. 2006;86: 89–154.
17. Forssberg H. Stumbling corrective reaction: a phase-dependent compensatory reaction during locomotion. J Neurophysiol. 1979;42: 936–953.
18. Eng JJ, Winter DA, Patla AE. Strategies for recovery from a trip in early and late swing during human walking. Exp Brain Res. 1994;102: 339–349. doi:10.1007/BF00227520
19. Thangal SNM, Donelan JM. Scaling of inertial delays in terrestrial mammals. PLoS One. 2020;15: e0217188. doi:10.1371/journal.pone.0217188
20. Ijspeert AJ, Daley MA. Integration of feedforward and feedback control in the neuromechanics of vertebrate locomotion: a review of experimental, simulation and robotic studies. Journal of Experimental Biology. 2023;226: jeb245784. doi:10.1242/jeb.245784
21. Miall RC, Weir DJ, Wolpert DM, Stein JF. Is the cerebellum a smith predictor? J Mot Behav. 1993;25: 203–216.
22. Garcia MS, Kuo AD, Peattie A, Wang P, Full RJ. Damping And Size: Insights And Biological Inspiration. International Symposium on Adaptive Motion of Animals and Machines. 2000.

23. Åström KJ, Murray RM. Feedback systems: an introduction for scientists and engineers. Princeton University Press. Princeton, New Jersey: Princeton University Press; 2008. Available: http://www.cds.caltech.edu/~murray/amwiki
24. Franklin GF, Powell JD, Emami-Naeini A. Feedback control of dynamic systems. Pearson Upper Saddle River, NJ; 2010.
25. Todorov E, Jordan MI. Optimal feedback control as a theory of motor coordination. Nat Neurosci. 2002;5: 1226–1235. doi:10.1038/nn963
26. Massion J. Movement, posture and equilibrium: Interaction and coordination. Prog Neurobiol. 1992;38: 35–56. doi:https://doi.org/10.1016/0301-0082(92)90034-C
27. De Groote F, Allen JL, Ting LH. Contribution of muscle short-range stiffness to initial changes in joint kinetics and kinematics during perturbations to standing balance: A simulation study. J Biomech. 2017;55: 71–77.
