# Supplementary material for: Effects of sensorimotor delays and muscle force capacity limits on the performance of feedforward and feedback control in animals of different sizes
Source: PLoS Comput Biol. 2026 Apr 21;22(4):e1012502. doi: 10.1371/journal.pcbi.1012502 (PMC13152214; doi:10.1371/journal.pcbi.1012502)
Supplement: Attachment — Submitted filename: PCOMPBIOL-D-24-re-review-v2.pdf [file pcbi.1012502.s003.pdf]

I appreciate the author's attempt to better define feedforward and feedback control and contextualize them better in the literature (e.g. in respect to Reviewer #1's comments). Although the authors do cite a good range of relevant research, more clarity is still required regarding what is meant by feedforward control. This was not addressed adequately in the response to reviewers, and the manuscript has not become clearer in this regard, unfortunately.

This is a significant conceptual issue in terms of framing the work, but that the paper's main contribution regarding modeling of feedback control remains valid. Nevertheless, it is essential to correct this in the next revision.

There are two (that I know of) standard notions of FF control in the literature. The introduction muddles them together along with "bang bang control" – all bang-bang controllers are not FF and all FF controllers are not bang-bang, so this is extremely confusing. The authors unclear discussions of control theory will unfortunately add confusion to the literature and obfuscate the contributions of the study. Below is my attempt to provide clear set of definitions that I sincerely hope will help the authors better frame their contributions. Something along these lines is necessary for me to recommend publication.

1) Classical FF control. In a traditional control system, when there is a reference trajectory, a feedforward controller maps the reference trajectory through a feedforward block that computes an input without waiting for self-motion feedback, and adds this to a feedback control term. This is well represented in Yamagami et al: https://www.sciencedirect.com/science/article/pii/S2405896319300278

a.

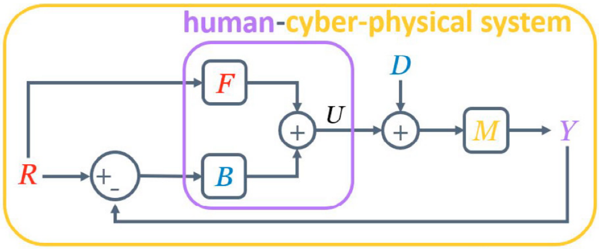

This diagram is cut from the Yamagami paper. (This is just one example paper and they certainly didn't invent the topic; but they do cite other papers therein that may be helpful.) Here, "F" is the feedforward block. Note that if there were no feedback ("B"), then the system dynamics would be Y = MF R + M D (in the Laplace domain, for a linear system). With feedback, F is still not included in the feedback term, namely Y = MFR + BM/(1+BM) R + M(/1+BM), i.e. the response to the disturbance changes with feedback, and a new response to the reference is added, but the "F" block only appears once in the MFR term. That is, the feedforward does not take any feedback into account. All this math is of course not needed in the paper, just adding it for tutorial purposes here.

This is the usual form of FF control, i.e. the one taught in an introductory control theory course. This formulation can be used when r(t) is measured in real time (as in the Yamagami paper or in this manuscript by Cao et al https://pubmed.ncbi.nlm.nih.gov/39990312/). The FF controller block "F" can also be applied to the case of creating a reference trajectory *r(t)* from a goal (e.g. https://doi.org/10.1093/brain/awu115). Given this feedforward reference, one computes the required joint torques to, in theory, perfectly produce the desired output. This is also known as either "computed-torque" control or "inverse dynamics" control, and is described in standard engineering textbooks such as Murray, Li, and Sastry's Introduction to Robotics. This can be easily formulated as follows: given a pre-computed movement (*not* a "perturbation"), compute the required muscle commands that in theory would exactly execute the trajectory; in robotics this computed torque control is typically augmented with a feedback term that measures error states.

In the diagram above from the Yamagami et al paper, the r(t) is not assumed to be known in real time. If it were, the "F" would "invert" the plant, i.e. "inverse dynamics". However, when r(t) is measured in real time, the F block is often a *partial inverse* (as the Buden paper and the Cao et al manuscript point out) that can be computed in real time. That said, it serves a very similar purpose to the inverse dynamics control scheme.

Using FF control to follow a predefined reference is also used extensively in the "optimal control" approaches in neuroscience, such as Wolpert's early eye saccade modeling among MANY others. This first notion of classic FF control is compatible with the author's references [16] and [33], and other examples, such as eye saccades, given in the manuscript. For example, [16] (Maisson et al): when a user has time to plan a multi-joint movement, some joints can create anticipatory forces that match the expected needs. NOTE: optimal control and FF control are not synonymous—Todorov and Jordan's famous paper used optimal control in a *feedback* control context, explicitly bucking the prevailing use of optimal control only for trajectory generation and open-loop (FF) control since most optimal control before then – and much since – has been FF control in the neuro literature.

2) Open-loop CPG. Another type of feedforward control is an open-loop CPG. The authors cite Full lab papers [14,15] in which the initial responses are mechanical; this is somewhat related to the idea of using a feedforward CPG played through a mechanical system that provides preflexive feedback – a CPG feeding an upright animal, versus a sprawled postural animal like a cockroach, would play out very, very differently due to different engagement of preflexes, but preflexes themselves are not "feedforward controllers" – the are rapid mechanical responses that serve a stabilizing role. The guinea fowl examples are also relevant here where FF control + preflex-like responses can reject perturbations without immediate neural feedback. This can be visualized using a classic diagram from the Holmes et al SIAM paper (https://doi.org/10.1137/S0036144504445133); the "bottom" examples in the diagram have FF

signals “down” to the mechanical system with no feedback.

230 HOLMES, FULL, KODITSCHEK, AND GUCKENHEIMER

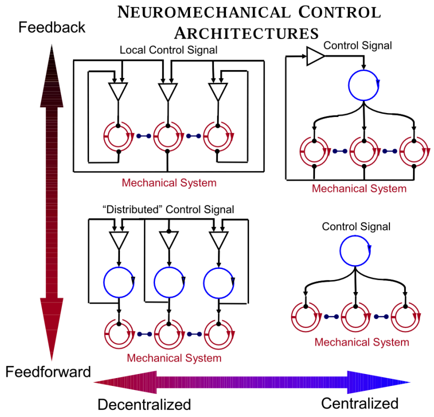

Fig. 7 *The schematic two-dimensional space of control architectures. As in Figure 5, single circles represent CPG oscillators, double circles represent mechanical oscillators such as limb components, and triangles represent neural control elements (analogous to operational amplifiers). Reprinted from* [208] *with permission from Elsevier.*

An example of feedforward control that exploits preflexes would be knowing that the platform you are standing on might move unexpectedly (such as on a subway car) and thus broadening your stance to be ready to passively absorb impulses. Along those lines Sefati et al (DOI: 10.1073/pnas.1309300110) show a feedforward strategy in which a fish partitions its fin into two inward waves (presumably using CPGs that drive the fin rays to generate the inward waves, and creating a passive damping-like term, potentially rejecting perturbations without feedback). Art Kuo’s simple models of a CPG with feedback to the CPG shows a way of thinking about the Holmes et all diagram as a Kalman filter with difference levels of sensory or motor noise; in those papers Kuo and colleagues argue for example that more sensory noise leads to weaker feedback and therefore more reliance on the FF aspects of the CPG.

The authors are right, here: “... running guinea fowl encountering obstacles maintain rhythmic feedforward control of proximal leg joints, while distal joints respond reflexively [9–11]. Humans adjust limb stiffness in anticipation of floor stiffness changes during hopping and running, relying on feedforward tuning” and “Fast, goal-directed ballistic movements, such as saccades and rapid reaching, also reflect feedforward-dominated control, where preplanned motor commands execute without time for feedback corrections [17].” It would be useful to clarify that preflexes are not “feedforward control” – as I think your manuscripts describe, feedforward control is a neural process, and those processes impact mechanical feedback (such as co-contracting makes muscle preflexes stiffer).

So, **those are the only two well documented notions of FF control that I know about**. Unfortunately, the manuscript muddle these two notions of control and their variations (e.g. known reference trajectory in advance or not, or inclusion of preflexes or not, etc) along with their own notion

of FF control: “The fastest neurally mediated control strategy to reject a perturbation is a type of feedforward control called “bang-bang control” [32,33]”. This statement is not meaningful for at least two reasons:

- a neurally mediated control strategy to reject a perturbation is a feedback strategy; a perturbation impacts the plant (e.g. initial conditions of the mechanical system), and thus should be thought about as the “D” input in the Yamagami diagram. Think of it this way: FF control (a la the framework used by Yamagami et al paper) is a response to an exteroceptive *sensory* measurements of “stuff out there” without using any self-motion feedback, while FB control is also a sensory response, but to self-motion feedback (it is often drawn using a comparator of the external reference, but it need not be; see the Cao et al paper Figure 9 compared to Figure 1). The author’s intuition that this can be a more aggressive response is not wrong, since measurement delays do not destabilize a feedforward response.
- a bang-bang controller isn’t necessarily feedforward. In fat, the paper cited in the manuscript about this is [32] (Rao et al); **they used bang-bang control as a feedback strategy in that paper,** underscoring my point of about this confusion. One will notice in Figure 12 of that paper that the minimum time control includes feedback delay.

The current manuscript then adds that FF control is *“... an open-loop, pre-planned motor command computed assuming perfect knowledge of the system and perturbation [34–36].”* This is also not a standard notion of FF. You don’t know perturbations in advance, if you knew them, why would there be a delay?

So, all of this is to say the manuscript does not disentangle these notions of FF control (the two above that are standard plus their own notion which is not really FF control) and conflate FF control with bang-bang control, which need not be a FF strategy.

Thus, the introduction needs to be rewritten in light of the above to clarify what FF control is. In terms of how to frame the “FF” controller in the paper, I don’t think this is a “CPG” type problem so the CPG framework of FF control is not the right notion. Instead, I would recommend placing the results in the Yamagami / Cao FF vs. FB diagram framework. (Note that Figure 1 of the present manuscript that shows FF control doesn’t fit any notion of FF control). To do this, one could think about a FF response to a reference signal, such as a cheetah chasing a gazelle, moth tracking a flower, or a runner following sidewalk (although the latter brings up notions of preview control; see Cao et al for more info). Note that the feedforward and feedback results could be described using the Yamagami-like or Cao-like diagram. The FF strategy is then a change in reference that is anticipated through a FF controller – or one could “anticipate” a disturbance D.

In sum, disentangling the notions of FF control in the intro, clarifying how preflexes fit in, and finally defining a clear notion of FF control for the paper in a way that comports with the literature is essential. (I do NOT think any substantive new research is required to do this.) Simply removing “feedforward

control" from the title, as done in this revision, does not resolve the core issues. The structural argument of the paper still relies on a description FF control that remains unmoored to the literature on FF control.

OTHER CONCERNS:

1.

Throughout the manuscript, the authors make overly deterministic statements without sufficient empirical evidence:

- "feedback control appears ineffective for terrestrial mammals of all sizes, as the fastest response times exceeded available movement times." Overly general!
- "These time delays make feedback control consistently too slow for rapid perturbation responses." This is a meaningless statement unless somehow you add that perturbations can be anticipated.
- "Feedback control does not seem effective for fast perturbation responses in animals of any size." Over general and large animals slip or receive other perturbations commonly in the real world and do not always fail.

Such blanket assertions are scientifically unjustified and detract from the credibility of the work. They ignore the diversity of feedback control strategies in animals, including biological compensatory mechanisms like reflex modulation in spinal circuits (which can be fast) and hierarchical control.

2.

Various comments on the control modeling:

The manuscript suffers from vague definitions and unexplained parameter choices. Key terms such as "deadtime," "sensorimotor time delay," and "2% threshold" are not rigorously defined. For instance:

What exactly is meant by "deadtime"? Is it equivalent to the total sensorimotor delay, or a subcomponent?

What does the "2% threshold" refer to — 2% of full movement or just 0 to the desired angle? Some FB controllers may have a small steady-state error; in this case, a system may never reach 2%. Or a longer timescale correction, like an integral controller, can achieve the rest.

The PD controller should be motivated better. Why not PID or adaptive controllers? This can be justified a few different ways. E.g. for a two state system (angle, angular velocity) PD control is

equivalent to state feedback. But some justification should be provided.

The tau_iso was set to 0.25 but it was not obvious that this would be dimensionally scaled with all the other parameters.

Also, note that FF and FB delay are not necessariliy the same; see Cao et al (referenced above).

3.

The modeling choices and simplifications are not always justified or critically evaluated. For example:

The two main experimental tasks (swing and posture) are reduced to highly similar pendulum models with gravity excluded. This abstraction may erase the essential biomechanical differences the tasks are meant to illustrate, such as the fact that smaller animals typically have more legs on the ground and sprawled posture.

And if the author ignore the gravity, the normalized system in two swing task and posture task will be almost the same with different parameter and input, and just gravity during the delay.

Only one perturbation magnitude is shown for swing task (e.g., 30°), with no indication of how generalizable the findings are.

Further, the authors claim that “response time is independent of movement size in feedback control if the controller gains are the same and the torques do not exceed force capacity limits”, “In the middle region, response times begin to increase gradually with force capacity limits, Scaling of feedback control response times in animals as positive torques are clipped by the force limits, but negative torques are not.” This is a conditionally true but overly narrow statement, and such limited-scope results should not be presented as general conclusions.

6.

The structure of the manuscript is confusing, and some of the narrative shifts appear to be introduced only to satisfy reviewer suggestions rather than to enhance clarity or logic. As stated by the authors:

“To improve clarity and avoid repetition, we have reversed the order of subsections within the Methods and Results sections.”

This reordering does not improve clarity and instead disrupts the logical progression of ideas.

7.

Several assumptions are presented without acknowledging their biological limitations. For example, the claim that larger animals have proportionally weaker muscles (due to greater segment mass) is not universally true and depends heavily on species, muscle architecture, and ecological context. The authors should clearly state that the models and assumptions are specific to terrestrial quadrupeds or mammals, rather than generalizing to all animals. Furthermore, the treatment of reflexes as "feedforward-like" is conceptually problematic — they are better described as passive control

8.

"Perhaps non-intuitively, response time is independent of movement size in feedback control if the controller gains are the same and the torques do not exceed force capacity limits—it takes the same amount of time to perform a small movement as a large movement."

This is a false statement for the systems of interest unless a small angle approximation is used. It is not counterintuitive for linear systems: linear (asymptotically stable) systems decay exponentially, i.e. certain percentage (which is the nature of "half lives" and such). Since settling time is based on a percentage, it follows obviously that the decay time (for the linearization) is invariant to (non-zero) initial condition magnitude. If you had defined an absolute scale, rather than 2%, then of course this scaling would follow what I think you are suggesting is the "intuitive" trend.

So, after clarification of small-angle approximation, this result shouldn't be described as a surprise, as it is not surprising. Rather, a simple clarification along the lines of the above will remove any confusion someone has about this.
