# Supplementary material for: Effects of sensorimotor delays and muscle force capacity limits on the performance of feedforward and feedback control in animals of different sizes
Source: PLoS Comput Biol. 2026 Apr 21;22(4):e1012502. doi: 10.1371/journal.pcbi.1012502 (PMC13152214; doi:10.1371/journal.pcbi.1012502)
Supplement: Attachment — Submitted filename: Scaling of feedback control-revision 2-Response.docx [file pcbi.1012502.s004.docx]

# Response to Reviewers

PCOMPBIOL-D-24-01586R1

Effects of sensorimotor delays and muscle force capacity limits on the performance of feedforward and feedback control in animals of different sizes

Sayed Naseel Mohamed Thangal, Heather L. More, C. David Remy and J. Maxwell Donelan
=====================================================================

Reviewer #1: Review PCOMPBIOL-D-24-01586R1

I appreciate the authors’ thorough and thoughtful responses to the questions and issues that I raised in my initial review, as well as the points raised by the other reviewer. Overall, the authors have done a very good job to improve the clarity and organization of their paper. The paper now reads much more clearly and is easier to follow the authors’ reasoning and modeling approach. I very much appreciate making clear the rationale for explaining why the presentation of the scaled models precedes the normalized feedback model in the methods but is reversed in the results. I also appreciate that more complex Hill-muscle models are not incorporated in the modeling approach given the lack of data across animal size. In summary, showing that differing control strategies for both tasks (swing and postural likely exist for small versus large animals, with the latter favoring feedback control whereas small animals that are overdamped can benefit from feedforward control for fast movements is a nice and valuable outcome of the authors’ modeling analysis.

Thank you for reviewing our changes and appreciating our research study. We have addressed your additional comments below.

I have only a few additional points to make/suggest:

l. 137: wrt the statement here “rendering their muscles proportionally weaker [26,38–41].” I would argue that Biewener, Science 1989 and/or Biewener, Science 1990, following from Alexander et al. [38], clearly noted the scale effects of size on muscle force capacity (matched to muscle x-area to maintain similar stresses across size), and should be included in this reference list. Also, I do not believe Heglund et al. 1979 Science [40] explicitly address the matter of relatively weaker muscles, in the context of examining SFreq scaling and A.V. Hill’s analysis of muscle power scaling. So, unclear why it is in the list.

Thank you for providing stronger references for the statement on muscle strength changes with animal size. We have added the new references and removed Heglund et al. 1974.

L. 161: suggest adding “a forelimb swing leg repositioning task.” and l. 162 “a hindlimb posture recovery task under the control of ankle…” to make it quite clear that two different limbs (and joints) are being modeled for the swing and postural control tasks.

Thank you for suggesting this clarification. We have modified the purpose paragraph as follows:

“Consequently, we quantified response times for two perturbation response tasks: a forelimb swing leg repositioning task under the control of shoulder muscles (swing task), and a posture recovery task under the control of distal muscles of all four legs (posture task). “

l. 283-288: I appreciate that Alexander et al. [38] only report data for the triceps muscle scaling – and yes, the long head of triceps does cross the shoulder to assist in shoulder flexion or retraction. HOWEVER, the moment arm reported by [38] for triceps is at the elbow joint, not the shoulder. This adds to the problematic approach of using triceps muscle scaling data for analysis of the shoulder swing task. This needs to be addressed and further clarified in revision; otherwise, readers would assume the moment arm scaling is for the shoulder, but not what [38] reports. In this context, I think you might consider a sensitivity analysis for the moment arm scaling relationship of what would be appropriate for shoulder muscles. Note that Biewener Science 1990 reports the scaling of antigravity muscle moment arms across forelimb joints for mammals of different size to be 0.44, slightly greater than 0.41 for the triceps at the elbow reported by Alexander et al [38]. In any event, you might consider how sensitive your results are to differing moment arm scaling relationships – perhaps 0.38 to 0.44, spanning 0.41 for your shoulder swing task model.

This is a great point. We have performed the sensitivity analysis on shoulder muscle moment arms and added a new paragraph to the Methods section with the sensitivity analyses. The Methods section 2.1.3 paragraph 2 now reads:

“We tested the sensitivity of our results to certain parameters. We used an exponent of 0.41 for the swing task moment arm based on data for the triceps muscle from Alexander et. al. 1981, due to a lack of scaling information for shoulder muscles in the literature. However, the cited moment arm data is for the triceps muscle about the elbow, not the shoulder. Biewener 1990 reported that antigravity muscle moment arms for forelimb muscles scale with 0.44 [1]. We varied muscle moment arm exponents from 0.38 to 0.44 while keeping movement size at 30° and studied its effects on response times. Feedback response times did not change, as the feedback controllers were not able to reach the muscle force capacity limits in either case. The coefficient of the scaling of feedforward response times changed by 0.65% to -0.53% for moment arm exponents of 0.38 to 0.44 respectively. The exponent of the scaling of feedforward response times changed by +3% to -3% for moment arm exponents of 0.38 to 0.44 respectively. These results suggest that our conclusions are not very sensitive to the exponent we use for shoulder muscle moment arm.”

===================================================================

Reviewer #2: Review uploaded as attachment.

Major comment:

I appreciate the author’s attempt to better define feedforward and feedback control and contextualize them better in the literature (e.g. in respect to Reviewer #1’s comments). Although the authors do cite a good range of relevant research, more clarity is still required regarding what is meant by feedforward control. This was not addressed adequately in the response to reviewers, and the manuscript has not become clearer in this regard, unfortunately.

This is a significant conceptual issue in terms of framing the work, but that the paper’s main contribution regarding modeling of feedback control remains valid. Nevertheless, it is essential to correct this in the next revision.

- Classical FF control
- Open loop CPG

There are two (that I know of) standard notions of FF control in the literature. The introduction muddles them together along with “bang bang control” – all bang-bang controllers are not FF and all FF controllers are not bang-bang, so this is extremely confusing. The authors unclear discussions of control theory will unfortunately add confusion to the literature and obfuscate the contributions of the study. Below is my attempt to provide clear set of definitions that I sincerely hope will help the authors

better frame their contributions. Something along these lines is necessary for me to recommend publication.

So, those are the only two well documented notions of FF control that I know about.

Unfortunately, the manuscript muddle these two notions of control and their variations (e.g. known reference trajectory in advance or not, or inclusion of preflexes or not, etc) along with their own notion of FF control: “The fastest neurally mediated control strategy to reject a perturbation is a type of feedforward control called “bang-bang control” [32,33]”. This statement is not meaningful for at least two reasons:

- a neurally mediated control strategy to reject a perturbation is a feedback strategy; a perturbation impacts the plant (e.g. initial conditions of the mechanical system), and thus should be thought about as the “D” input in the Yamagami diagram. Think of it this way: FF control (a la the framework used by Yamagami et al paper) is a response to an exteroceptive *sensory* measurements of “stuff out there” without using any self-motion feedback, while FB control is also a sensory response, but to self-motion feedback (it is often drawn using a comparator of the external reference, but it need not be; see the Cao et al paper Figure 9 compared to Figure 1). The author’s intuition that this can be a more aggressive response is not wrong, since measurement delays do not destabilize a feedforward response.
- A bang-bang controller isn’t necessarily feedforward. In fat, the paper cited in the manuscript about this is [32] (Rao et al); they used bang-bang control as a feedback strategy in that paper, underscoring my point of about this confusion. One will notice in Figure 12 of that paper that the minimum time control includes feedback delay.
- The current manuscript then adds that FF control is *“… an open-loop, pre-planned motor command computed assuming perfect knowledge of the system and perturbation [34–36].”* This is also not a standard notion of FF. You don’t know perturbations in advance, if you knew them, why would there be a delay?

So, all of this is to say the manuscript does not disentangle these notions of FF control (the two above that are standard plus their own notion which is not really FF control) and conflate FF control with bang-bang control, which need not be a FF strategy.

Thus, the introduction needs to be rewritten in light of the above to clarify what FF control is. In terms of how to frame the “FF” controller in the paper, I don’t think this is a “CPG” type problem so the CPG framework of FF control is not the right notion. Instead, I would recommend placing the results in the Yamagami / Cao FF vs. FB diagram framework. (Note that Figure 1 of the present manuscript that shows FF control doesn’t fit any notion of FF control). To do this, one could think about a FF response to a reference signal, such as a cheetah chasing a gazelle, moth tracking a flower, or a runner following sidewalk (although the latter brings up notions of preview control; see Cao et al for more info). Note that the feedforward and feedback results could be described using the Yamagami-like or Cao-like diagram. The FF strategy is then a change in reference that is anticipated through a FF controller – or one could “anticipate” a disturbance D.

In sum, disentangling the notions of FF control in the intro, clarifying how preflexes fit in, and finally defining a clear notion of FF control for the paper in a way that comports with the literature is essential.

(I do NOT think any substantive new research is required to do this.) Simply removing “feedforward control” from the title, as done in this revision, does not resolve the core issues. The structural argument of the paper still relies on a description FF control that remains unmoored to the literature on FF control.

Thank you for taking the time to meet with us and as well as providing us with a detailed explanation on the types of feedforward control. This clarified how our notion of feedforward control and bang-bang control differs from convention.

We have updated our descriptions of feedback and feedforward control based on your review:

- We now clarify feedforward (FF) vs feedback (FB) using the Yamagami/Cao framework: A perturbation to the body triggers a perturbation response from the neural controllers after an initial deadtime. FF issues motor commands using a minimum time implementation without using self-state to regulate the commands; FB uses self-state to constantly adjust the motor commands to reduce error to a reference target.
- We now use consistent terminology (“reference target”) across FF and FB and state explicitly that both controllers receive the same reference target.
- We have now removed the implication that FF requires knowing the disturbance in advance; FF is triggered by a perturbation and proceeds without self-state feedback.
- We have now moved CPG/preflex material out of the Introduction and into the Discussion as biological context.

We have changed our description of bang-bang control based on your review:

- We have now removed bang-bang as part of the FF definition in the Introduction; it is treated solely as a minimum-time implementation.
- We provide further details about bang-bang actuation in the Methods, and clarify that the FF controller does not use self-state sensory feedback.

We have updated the Introduction with the following changes:

Introduction paragraph 1 now reads:

“Animals rely on both feedforward and feedback control strategies to respond to perturbations they encounter when moving through the world. Consider an animal whose foot gets caught on a vine, requiring a quick perturbation response to prevent a fall and injury. Sensors (cutaneous, proprioceptive, vestibular etc.) would detect the perturbation and transmit the signal through afferent nerves to neural controllers in the spinal cord and brain. These neural controllers could then trigger a stumble corrective response or elevation strategy that involves lifting and swinging the foot forward to a new position or “reference target” [2,3]. The neural controllers could use either feedforward control or feedback control to determine the muscle activation (motor) commands to perform the perturbation response. Feedback control takes in sensory information about the body’s movements and compares it to the desired reference target throughout the response. The controller uses this changing error signal to generate the motor commands that accurately reposition the body to the new stable position [4,5]. In contrast, feedforward control uses predefined motor commands that reposition body segments to the reference target without continuously updating its response based on sensory feedback [6–8]. One benefit of this strategy is that it can be used to rapidly reject a perturbation as it does not have to wait for continuous adjustment by sensory feedback.”

The initial part of Introduction paragraph 2 now reads:

“In reality, animals use neural controllers that simultaneously leverage feedforward and feedback control, perhaps to benefit from their individual advantages while compensating for each other’s drawbacks [8–10]. Computational models developed to study animal movement often implement a control system with simultaneous feedforward and feedback strategies [11–13]. Once a perturbation occurs, neural controllers are triggered which determine a reference target that the body (plant) has to be repositioned to in order to regain stability. A feedforward controller then uses predetermined motor commands to reposition body segments to the reference target, without using sensory feedback. A feedback controller works in parallel, generating motor commands based on the error between the same reference target and sensory feedback, addressing any deviations which were not predicted by the feedforward controller. Neural controllers might prioritize feedforward or feedback control depending on factors such as the body segments being moved, time available and sensory information [8,11].”

Introduction paragraph 3 now includes an updated description of feedforward minimum time control:

“The fastest neurally mediated control strategy to reject a perturbation is equivalent to feedforward control with a minimum-time implementation [14,15]. It is an open-loop, pre-planned motor command without using sensory feedback [16–18]. As such, it represents the theoretical upper limit of response speed achievable by a control strategy, constrained only by initial sensorimotor delay, actuator force capacity, and system dynamics.”

We have removed the statement *“given perfection knowledge of the perturbation and system dynamics”* from our descriptions of feedforward control in the Introduction.

We have updated Figure 1 to show a reference target for both FF and FB control.

We have updated the caption of Figure 1 with improved descriptions:

“The perturbation response is triggered by an external event such as a trip or a push to the body. The body segments are represented here by the plant using rigid body dynamics and pendular models. The sensorimotor delay block represents all the time delays in the neural control pathways from detection of the perturbation, transmission through sensory and motor nerves, to synaptic computations and activating muscles. Once the neural controllers receive sensory information about the perturbation, they would determine a perturbation response that involves repositioning the body to a desired reference target. The feedback system (top) uses a proportional-derivative (PD) controller to generate torque based on time-delayed state feedback. The feedforward system (bottom) optimizes controller torques to minimize response time resulting in bang-bang actuation, serving as an upper-bound benchmark for the fastest possible perturbation response. The force capacity limits block restrict the maximum positive and negative torques that can be applied by the controller, based on the force capacity of the muscles used in the task.”

We updated our description of feedforward control in Methods section 2.1.1 to read:

“Under feedforward control, the plant dynamics are identical, but the actual torque ($\tau_{act}$) is found by an optimization to minimize the time required to complete the movement; this results in bang-bang actuation—a control strategy that switches instantaneously between our estimates of the maximum isometric flexion and extension torques [15–18].”

**“**The feedfoward controller does not use self-state sensory information; we determine $t_{switch}$ by optimizing for fastest response time.**”**

We have moved the description of studies that involve CPG+preflexes to Discussion paragraph 3, which now reads:

“While we have modeled our perturbation responses as goal-directed repositioning movements, perturbation responses during rhythmic locomotion also involve spinal neural oscillators and passive mechanisms. Preflexes are passive viscoelastic responses to perturbations that act instantaneously and without neural input, although they are modulated by the active state of muscles and joint positions during movement [19]. Running guinea fowl encountering obstacles maintain rhythmic feedforward control of proximal leg joints, while distal joints respond preflexively [20–22]. Humans adjust limb stiffness in anticipation of floor stiffness changes during hopping and running, relying on feedforward tuning and preflexive responses [23,24]. And cockroaches subjected to impulsive lateral perturbations generate peak ground reaction forces far faster than can be explained by neural reflexes, highlighting how intrinsic mechanical properties and pre-tuned musculoskeletal dynamics can mediate instantaneous responses [25,26]. Kuo 2002 described how central pattern generators and reflex circuits work together to form a state estimator which can optimally compensate for both disturbances and sensorimotor noise [8]. We have not modeled these CPG and preflexive control mechanisms, and considered only upright terrestrial quadrupedal mammals, to simplify modeling choices.”

Minor comments:

1. Throughout the manuscript, the authors make overly deterministic statements without sufficient empirical evidence:

A. “feedback control appears ineffective for terrestrial mammals of all sizes, as the fastest

response times exceeded available movement times.” Overly general!

B. “These time delays make feedback control consistently too slow for rapid perturbation

responses.” This is a meaningless statement unless somehow you add that perturbations can be anticipated.

C. “Feedback control does not seem effective for fast perturbation responses in animals of any size.” Over general and large animals slip or receive other perturbations commonly in the real world and do not always fail.

Such blanket assertions are scientifically unjustified and detract from the credibility of the work. They ignore the diversity of feedback control strategies in animals, including biological compensatory mechanisms like reflex modulation in spinal circuits (which can be fast) and hierarchical control.

We appreciate your feedback on our statements that came across as overly general. We have reviewed and corrected overly deterministic statements, and now constrain broad claims to the studied context: rapid perturbation responses with tight time budgets in terrestrial mammals (e.g., stride-scale recovery during fast running/postural balance), avoiding “all sizes/any perturbation” generalizations.

2. Various comments on the control modeling:

The manuscript suffers from vague definitions and unexplained parameter choices. Key terms such as “deadtime,” “sensorimotor time delay,” and “2% threshold” are not rigorously defined.

A: For instance: What exactly is meant by “deadtime”? Is it equivalent to the total sensorimotor delay, or a subcomponent?

B: What does the “2% threshold” refer to — 2% of full movement or just 0 to the desired angle? Some FB controllers may have a small steady-state error; in this case, a system may never reach 2%. Or a longer timescale correction, like an integral controller, can achieve the rest.

C: The PD controller should be motivated better. Why not PID or adaptive controllers? This can be justified a few different ways. E.g. for a two state system (angle, angular velocity) PD control is equivalent to state feedback. But some justification should be provided.

D: The tau_iso was set to 0.25 but it was not obvious that this would be dimensionally scaled with all the other parameters.

E: Also, note that FF and FB delay are not necessarily the same; see Cao et al (referenced above).

Thank you for pointing out definitions and parameter choices that need improvement.

A. We have clarified that sensorimotor time delays represent all the delays in the neural control pathways and that the deadtime is equal to the entire sensorimotor delay time in Methods section 2.1.1 **Feedback controller** paragraph.

*“The sensorimotor delay represents all the time delays in the neural control pathways including the detection of the perturbation by sensors (proprioceptive, cutaneous, vestibular), transmitting the signals through the sensory and motor nerves, computing perturbation responses at spinal synapses, and activating and generating muscle forces* [27]*. We implemented an initial deadtime at the start of the perturbation response equal to the sensorimotor delay; during which the controller does not apply any forces, and the forelimb moves under the influence of gravity. Once this deadtime is over, the controller begins to exert forces on the pendulum, using time-delayed state information.”*

B. We have clarified that the 2% threshold is for the entire 30° movement in **Simulations and optimization – feedback control** paragraph in Methods section 2.1.1:

*“We quantified response time as the settling time with 2% thresholds of the entire movement from the initial position to the reference target (*$\theta_{r}$*) (Fig 3b).”*

As the PD controller alone could not reach the reference target due to the effects of gravity on the plant, we compared the use of a PID controller vs. a PD controller with a steady-state term. We describe this in the **Simulations and optimization – feedback control** paragraph in Methods section 2.1.1:

“To counter the steady state gravitational torques at the target position of +15°, we explored the use of a steady state torque ($\tau_{steadystate}$), or integral control. We used the steady state torque option because it required fewer parameters to optimize and produced faster response times.”

We now report robustness of conclusions to the settling threshold (1%, 2%, 4%) and note that 2% is conventional but arbitrary. In Methods section 2.1.3, we have added a paragraph with sensitivity analyses to the settling time threshold:

“We tested the sensitivity of our results to certain parameters. Our choice of 2% for settling time threshold, while arbitrary, is conventional in control theory and allows us to be consistent in determining response times across animal size. A tighter tolerance of 1% resulted in the coefficient in the scaling of swing task feedback response times for a 30° movement increasing by 75%, while a looser tolerance of 4% resulted in the response time coefficient decreasing by 8%; the exponent remained largely unchanged.”

C. In Discussion paragraph 2, we have improved our justification on why we use PD control including your suggestion.

“We used a proportional-derivative feedback controller as it represents full state feedback in a mechanical system. It is simple to implement, simple to interpret, and has been successfully used in the literature to model control of locomotion [28–32]. The actual control strategies implemented within the spinal reflex pathways are undoubtedly more complex, involving several control pathways with varying time delays, and remains a topic of research [22,33–35].”

D. We have provided the normalization of $\tau_{iso}$ in section 2.2 to clarify that it gets scaled with the parameters for each animal size:

“The force capacity, represented by $\overline{\tau}_{iso}=\frac{\tau_{iso}}{\left( \frac{I\theta_{r}}{{t_{SM}}^{2}} \right)}$, is the only free parameter in this model.”

E. Thank you for pointing us to this interesting study. We have referenced these results in Methods section 2.1.3:

*“We also simplified the scaled feedback models by assuming that all sensorimotor delays occur in the feedback (sensory) pathway. In real life, there are delays in both the feedback (sensory) pathway and the feedforward (motor) pathway. In the supplementary materials section S1, we show how this assumption does not affect outcomes for the step response inputs we use in our simulations. While we have assumed that the feedforward and feedback control systems experience the same total sensorimotor delays, Cao et. al. 2025 show that the neural controllers might face different synaptic (computational) delay times when using feedforward vs. feedback control* [12]*.”*

3. The modeling choices and simplifications are not always justified or critically evaluated. A. For example: The two main experimental tasks (swing and posture) are reduced to highly similar pendulum models with gravity excluded. This abstraction may erase the essential biomechanical differences the tasks are meant to illustrate, such as the fact that smaller animals typically have more legs on the ground and sprawled posture.

B. And if the author ignore the gravity, the normalized system in two swing task and posture task will be almost the same with different parameter and input, and just gravity during the delay.

C. Only one perturbation magnitude is shown for swing task (e.g., 30°), with no indication of how generalizable the findings are.

D. Further, the authors claim that “response time is independent of movement size in feedback control if the controller gains are the same and the torques do not exceed force capacity limits”，“In the middle region, response times begin to increase gradually with force capacity limits, as positive torques are clipped by the force limits, but negative torques are not.” This is a conditionally true but overly narrow statement, and such limited-scope results should not be presented as general conclusions.

Thank you for providing clear suggestions on improving our description of modeling choices and simplifications.

A. We reduced our models to the simplest systems possible to study the effects of sensorimotor delays and muscle force capacity limits on neural control. We acknowledge that the simplifications miss several features that distinguish small and large animals in Discussion paragraph 2:

“Although animal limbs are multi-jointed and multi-muscled, we used pendular models to represent body segments, and a single pair of opposing muscles to actuate them [36,37]. We have also not considered several animal features that have been shown to change with animal size such as posture, limb stiffness, joint damping and sensor accuracy; evaluating the effects of these features would require more complete neuromusculoskeletal models [38–40].”

B. The normalized model does ignore gravity, making the swing and posture task single degree of freedom rotational systems which differ only in initial conditions. In supplementary materials section S3, we describe how the two tasks result in different optimal controller gains, and behaviour in the force capacity limits vs. response time analyses.

C. In Methods section 2.1.3, we have added a paragraph with sensitivity analyses to the movement size:

“We tested the sensitivity of our results to certain parameters. To check sensitivity to movement size, we evaluated response times in the swing task for the extreme case of a 180° movement size. The feedback response time coefficient reduced by 6%, while the exponent did not change. This matches predictions that PD control response times are not sensitive to movement size (Methods section 2.1.1), with small deviations due to the effects of steady state torque, initial deadtime and gravity. In contrast, the swing task feedforward response time coefficient increased by 66.4%, while the exponent increased by 3.7%, for the 180° repositioning.”

D. We have addressed the concern about the first sentence in our response to comment 6. We clarify that the second sentence is stated only regarding the results of the normalized model shown in Fig 2.

4. The structure of the manuscript is confusing, and some of the narrative shifts appear to be introduced only to satisfy reviewer suggestions rather than to enhance clarity or logic. As stated by the authors:

“To improve clarity and avoid repetition, we have reversed the order of subsections within the Methods and Results sections.”

This reordering does not improve clarity and instead disrupts the logical progression of ideas.

We recognize how the wording might be misunderstood to indicate that we changed the organization after the review. We have not changed the order of subsections after the review; it was already in the flipped order in our original manuscript. We clarified the reason for the flipped organization to Reviewer 1 as he had noticed figures being cited out of order. We have reworded paragraph 2 of the Methods section to be more clear.

“To improve clarity and avoid repetition, we present subsections in different orders within the Methods and Results sections.”

5. Several assumptions are presented without acknowledging their biological limitations. For example, the claim that larger animals have proportionally weaker muscles (due to greater segment mass) is not universally true and depends heavily on species, muscle architecture, and ecological context. The authors should clearly state that the models and assumptions are specific to terrestrial quadrupeds or mammals, rather than generalizing to all animals. Furthermore, the treatment of preflexes as “feedforward-like” is conceptually problematic — they are better described as passive control.

We have updated the text to address these concerns:

The relevant sentence in Introduction paragraph 5 now reads:

“Considering cursorial terrestrial mammals, larger animals generally have larger and stronger muscles when compared to smaller animals; however their body segments are also heavier rendering their muscles proportionally weaker [1,27,36,41–43].”

We have also removed the term “feedforward like” while describing preflexes in the paragraph that used to be in the Introduction, but has now been moved to paragraph 3 of the Discussion.

6. “Perhaps non-intuitively, response time is independent of movement size in feedback control if the controller gains are the same and the torques do not exceed force capacity limits—it takes the same amount of time to perform a small movement as a large movement.”

This is a false statement for the systems of interest unless a small angle approximation is used. It is not counterintuitive for linear systems: linear (asymptotically stable) systems decay exponentially, i.e. certain percentage (which is the nature of “half lives” and such). Since settling time is based on a percentage, it follows obviously that the decay time (for the linearization) is invariant to (non-zero) initial condition magnitude. If you had defined an absolute scale, rather than 2%, then of course this scaling would follow what I think you are suggesting is the “intuitive” trend.

So, after clarification of small-angle approximation, this result shouldn’t be described as a surprise, as it is not surprising. Rather, a simple clarification along the lines of the above will remove any confusion someone has about this.

Thank you for providing this clear explanation for why PD control is invariant to movement size. We have incorporated your explanation into our revised version of the **Perturbations** paragraph in Methods section 2.1.1:

“**Perturbations**. In feedforward control responses, the response time depends on the size of the movement—small movements are accomplished quickly, but large movements take more time. Response time is independent of movement size in a linear PD control system (without nonlinearities like gravity or saturation limits) if the controller gains are not changed—small and large movements take the same amount of time. Linear PD control systems decay exponentially, moving a certain percentage of the movement to the reference target in a given amount of time. As settling time is also defined as a percentage of the reference target, the response time becomes invariant to movement size [44]. Larger movements create larger error terms (Eqn 4), resulting in larger controller torques that reposition the body within the same amount of time. While our feedback control system does consider nonlinear behaviour (gravity, saturation limits, steady state torque and initial deadtime), the linear PD control behaviour dominates. This results in feedback response times changing very little with movement size. Thus, to compare response times in the two types of control, it is useful to pick a movement size. Here we used a swing leg repositioning from -15° to +15° because sensorimotor delays and inertial delays are equally matched in a one kg animal for this movement size [41].”

## References

1.	Biewener AA. Biomechanics of mammalian terrestrial locomotion. Science (1979). 1990;250: 1097–1103. doi:10.1126/science.2251499

2.	Eng JJ, Winter DA, Patla AE. Strategies for recovery from a trip in early and late swing during human walking. Exp Brain Res. 1994;102: 339–349. doi:10.1007/BF00227520

3.	Rossignol S, Dubuc R, Gossard J-PP. Dynamic Sensorimotor Interactions in Locomotion. Physiol Rev. 2006;86: 89–154.

4.	Madhav MS, Cowan NJ. The synergy between neuroscience and control theory: the nervous system as inspiration for hard control challenges. Annu Rev Control Robot Auton Syst. 2020;3: 243–267.

5.	Cowan NJ, Ankarali MM, Dyhr JP, Madhav MS, Roth E, Sefati S, et al. Feedback control as a framework for understanding tradeoffs in biology. Integrative and Comparative Biology. 2014. pp. 223–237. doi:10.1093/icb/icu050

6.	Desmurget M, Grafton S. Forward modeling allows feedback control for fast reaching movements. Trends in Cognitive Sciences. 2000. pp. 423–431. doi:10.1016/S1364-6613(00)01537-0

7.	Wolpert DM, Kawato M. Multiple paired forward and inverse models for motor control. Neural Networks. 1998. pp. 1317–1329. doi:10.1016/S0893-6080(98)00066-5

8.	Kuo AD. The relative roles of feedforward and feedback in the control of rhythmic movements. Motor Control. 2002;6: 129–145. doi:10.1123/mcj.6.2.129

9.	Dickinson MH, Farley CT, Full RJ, Koehl MAR, Kram R, Lehman S. How Animals Move: An Integrative View. Science (1979). 2000;288: 100–106. doi:10.1126/science.288.5463.100

10.	Gordon JC, Holt NC, Biewener A, Daley MA. Tuning of feedforward control enables stable muscle force-length dynamics after loss of autogenic proprioceptive feedback. Elife. 2020;9: e53908. doi:10.7554/eLife.53908

11.	Yamagami M, Howell D, Roth E, Burden SA. Contributions of feedforward and feedback control in a manual trajectory-tracking task. IFAC-PapersOnLine. 2019;51: 61–66.

12.	Cao D, Wilkinson MGT, Bastian AJ, Cowan NJ. Feedback and feedforward control are differentially delayed in cerebellar ataxia. bioRxiv. 2025.

13.	Bhanpuri NH, Okamura AM, Bastian AJ. Predicting and correcting ataxia using a model of cerebellar function. Brain. 2014;137: 1931–1944.

14.	Rao VG, Bernstein DS. Naive control of the double integrator. IEEE Control Systems Magazine. 2001;21: 86–97. doi:10.1109/37.954521

15.	Nelson WL. Physical principles for economies of skilled movements. Biol Cybern. 1983;46: 135–147.

16.	Libby T, Johnson AM, Chang-Siu E, Full RJ, Koditschek DE. Comparative Design, Scaling, and Control of Appendages for Inertial Reorientation. IEEE Transactions on Robotics. 2016;PP. doi:10.1109/TRO.2016.2597316

17.	Tsianos GA, Rustin C, Loeb GE. Mammalian muscle model for predicting force and energetics during physiological behaviors. IEEE Transactions on Neural Systems and Rehabilitation Engineering. 2012;20: 117–133. doi:10.1109/TNSRE.2011.2162851

18.	Wong JD, Bobbert MF, Van Soest AJ, Gribble PL, Kistemaker DA. Optimizing the distribution of leg muscles for vertical jumping. PLoS One. 2016;11. doi:10.1371/journal.pone.0150019

19.	Brown I, Loeb G. A reductionist approach to creating and using neuromusculoskeletal models. Biomechanics and neural control of posture and movement. 2000; 148–163. doi:10.1007/978-1-4612-2104-3_10

20.	Daley MA, Biewener AA. Running over rough terrain reveals limb control for intrinsic stability. Proceedings of the National Academy of Sciences. 2006;103: 15681–15686. doi:10.1073/pnas.0601473103

21.	Daley MA, Usherwood JR, Felix G, Biewener AA. Running over rough terrain: guinea fowl maintain dynamic stability despite a large unexpected change in substrate height. Journal of experimental biology. 2006;209: 171–187.

22.	Nishikawa K, Biewener AA, Aerts P, Ahn AN, Chiel HJ, Daley MA, et al. Neuromechanics: An integrative approach for understanding motor control. Integr Comp Biol. 2007;47: 16–54. doi:10.1093/icb/icm024

23.	Farley CT, Gonzalez O. Leg stiffness and stride frequency in human running. J Biomech. 1996;29: 181–186.

24.	Ferris DP, Louie M, Farley CT. Running in the real world: adjusting leg stiffness for different surfaces. Proc R Soc Lond B Biol Sci. 1998;265: 989–994.

25.	Jindrich DL, Full RJ. Dynamic stabilization of rapid hexapedal locomotion. Journal of Experimental Biology. 2002;205: 2803–2822.

26.	Dudek DM, Full RJ. Passive mechanical properties of legs from running insects. Journal of Experimental Biology. 2006. doi:10.1242/jeb.02146

27.	More HL, Donelan JM. Scaling of sensorimotor delays in terrestrial mammals. Proceedings of the Royal Society B: Biological Sciences. 2018;285: 20180613. Available: http://dx.doi.org/10.1098/rspb.2018.0613

28.	Hatz K, Mombaur K, Donelan JM. Control of ankle extensor muscle activity in walking cats. J Neurophysiol. 2012;108: 2785–2793. doi:10.1152/jn.00944.2011

29.	Donelan JM, Pearson KG. Contribution of Force Feedback to Ankle Extensor Activity in Decerebrate Walking Cats. J Neurophysiol. 2004;92: 2093–2104. doi:10.1152/jn.00325.2004

30.	Geyer H, Seyfarth A, Blickhan R. Positive force feedback in bouncing gaits? Proc R Soc Lond B Biol Sci. 2003;270: 2173–2183.

31.	Prochazka A, Gillard D, Bennett DJ. Positive force feedback control of muscles. J Neurophysiol. 1997;77: 3226–3236.

32.	Welch TDJ, Ting LH. A feedback model reproduces muscle activity during human postural responses to support-surface translations. J Neurophysiol. 2008. doi:10.1152/jn.01110.2007

33.	Yakovenko S, Gritsenko V, Prochazka A. Contribution of stretch reflexes to locomotor control: A modeling study. Biol Cybern. 2004;90: 146–155. doi:10.1007/s00422-003-0449-z

34.	Zehr EP, Stein RB. What functions do reflexes serve during human locomotion? Prog Neurobiol. 1999;58: 185–205. doi:10.1016/S0301-0082(98)00081-1

35.	Prochazka A, Gillard D, Bennett DJ. Implications of positive feedback in the control of movement. J Neurophysiol. 1997. doi:10.1152/jn.1997.77.6.3237

36.	Alexander RM, Jayes AS, Maloiy GMO, Wathuta EM. Allometry of the leg muscles of mammals. J Zool. 1981;194: 539–552. doi:10.1111/j.1469-7998.1981.tb04600.x

37.	Böhmer C, Theil J-C, Fabre A-C, Herrel A. Atlas of Terrestrial Mammal Limbs. Boca Raton: CRC Press; 2020.

38.	Biewener AA. Biomechanical consequences of scaling. Journal of experimental biology. 2005;208: 1665–1676. doi:10.1242/jeb.01520

39.	Garcia MS, Kuo AD, Peattie A, Wang P, Full RJ. Damping And Size: Insights And Biological Inspiration. International Symposium on Adaptive Motion of Animals and Machines. 2000.

40.	More HL, Hutchinson JR, Collins DF, Weber DJ, Aung SKH, Donelan JM. Scaling of sensorimotor control in terrestrial mammals. Proceedings of the Royal Society B: Biological Sciences. 2010;277: 3563–3568.

41.	Thangal SNM, Donelan JM. Scaling of inertial delays in terrestrial mammals. PLoS One. 2020;15: e0217188. doi:10.1371/journal.pone.0217188

42.	Kilbourne BM, Hoffman LC. Scale effects between body size and limb design in quadrupedal mammals. PLoS One. 2013;8: e78392.

43.	Biewener AA. Scaling body support in mammals: limb posture and muscle mechanics. Science (1979). 1989;245: 45–48.

44.	Nise NS. Control Systems Engineering. 6th ed. John Wiley & Sons Inc.; 2011.
