# Supplementary material for: Effects of sensorimotor delays and muscle force capacity limits on the performance of feedforward and feedback control in animals of different sizes
Source: PLoS Comput Biol. 2026 Apr 21;22(4):e1012502. doi: 10.1371/journal.pcbi.1012502 (PMC13152214; doi:10.1371/journal.pcbi.1012502)
Supplement: Attachment — Submitted filename: Scaling of feedback control-rev 3-Response to reviewers.docx [file pcbi.1012502.s005.docx]

# Response to Reviewers

PCOMPBIOL-D-24-01586R2

Effects of sensorimotor delays and muscle force capacity limits on the performance of feedforward and feedback control in animals of different sizes

Sayed Naseel Mohamed Thangal, Heather L. More, C. David Remy and J. Maxwell Donelan
=====================================================================

Reviewer #1: 

Reviewer #1: The authors have addressed my minor concerns, compared with those raised by Reviewer #2. I will leave it to Reviewer 2, who's expertise in controls engineering related to neural sensory-motor control is considerably greater than mine. The authors have appeared to respond in depth to the several critical points of Rev #2.

Thank you for your positive review of our updated manuscript.

===================================================================

Reviewer #2:

I think the authors have addressed the major concerns raised in the previous review. The intro is now clear and comprehensive. The manuscript is clear and detailed, especially with the Supplementary Material. Our remaining concerns are minor.

Thank you for reviewing our manuscript in detail and strong recommendations to improve the clarity of our paper. We have addressed your new comments below.

1. In Figure 1, I don’t understand why the delay is placed in the bottom branch (feedback pathway); I think it is much more natural in the top branch of the feedback loop; this may seem counterintuitive, but the error signal is a combined measure of the perception of the reference target (which cannot occur without a delay) minus the perception of self movement (which also has a delay). If you put separate delays on both the reference and the self-movement feedback, each delay occuring "before" the “subtraction bubble”, and assume they are identical delays, then that is equivalent to a single delay after the subtraction bubble. (Note the reference delay and the feedback delay may not be identical, but it in a simplified model, lumping them together is reasonable.) However, the current figure compares an *un-delayed* reference measurement to *delayed* self-movement measurement. Note that writing it this way, with the delay in the top branch (capturing both the delay in feedback and the delay in the measured reference) allows one to lump the sensory delay and the motor delay, since they are all in the top branch and for a linear, single-input—single-output system, the blocks commute in a linear path like this. I don’t think this will change the results (and did not dig carefully enough back into the simulations to see if a change in how the reference jumps and is sensed would matter in the FB controller). It certainly will not improve the FB controller and in fact may make it slightly worse (same stability margins, maximum gains, etc, but perhaps slightly worse performance due to having to wait to sense the change in reference).

Thank you for bringing up this point. We have moved the sensorimotor time delay block to the top branch in Fig 1, and also updated other figures in the paper to reflect this change.

As we have used only a single delay block to simplify our feedback model, we had to choose between placing the delay in the top branch (feedforward or motor pathway) and bottom branch (feedback or sensory pathway). We had originally placed it in the bottom branch to emphasize to the reader that the feedback controller is making decisions based on time delayed sensory feedback.

We only consider a constant reference value, and the analysis we performed in Supplementary materials section S1 shows that we can place the time delay in either the top or bottom branch without affecting our results. We agree with your point that comparing a time-delayed state feedback signal to a non-time-delayed reference signal might confuse readers, and have updated the figure based on your suggestion.

Fig 1

Fig S3

2. Reference suggestion: I the sentence “Empirical studies by Horak and colleagues (1996) further showed that human postural responses dynamically reweight sensory feedback sources depending on context and perturbation characteristics [16]” I strongly recommend considering work by Kiemel et al., especially the following:

Kiemel T, Zhang Y, Jeka JJ: Identification of neural feedback for upright stance in humans: stabilization rather than sway minimization. J Neurosci 2011, 31:15144-15153.

Thank you for suggesting this research work. We have referenced and cited their work in L97-100 of the Introduction:

“Kiemel and colleagues conducted experiments to perform system identification of the musculoskeletal and feedback components involved in the neural control of upright posture, and their results are consistent with a feedback controller that aims to reduce body sway while using minimal muscle activation [17,18].”

3. Line 127, the statement that an FB controller must use lower gains to remain stable is not generally true. Reducing gains is just a common choice for controllers without delay compensation, but it is not a necessary outcome (e.g. with prediction /compensation, different architectures, etc). In other words, “must” is a bit too strong, although it is “typically true”. This could be softened in a reasonable way, e.g. “To compensate for long feedback delays, a feedback-delayed controller often requires lower loop gain above a stability-limiting frequency to remain stable when compared to a controller with shorter delays.”

You are correct. We have updated our wording.

4. Figure 1 (minor, can ignore if desired), the input torque only reaching saturation in positive limit could be confusing. Perhaps little dashed lines at “Fmax / Fmin” in both the force capacity limits box and in the little inset graphs as this would show that the FB controller just barely stays within the negative limit. Also, it is a bit weird that the FB controller is a deadbeat controller that finishes in finite time, but your paper uses a PD controller (asymptotic).

Thank you for pointing this out. We had shown representative torque lines to give the reader a preview of the behavior of our controllers. We have updated the representative FB and FF torque line in Fig 1 with saturation limits, and profiles that are closer to the real shapes from our test results. We also updated the FB torque line to show a more asymptotic response (see response to comment 1 above).

The flat region in the feedback motor torque is not due to the torque reaching the saturation limits, but due to the initial deadtime. As the controller has not received any state feedback during the initial deadtime, the PD controller computes torque based on this constant angle and 0 angular velocity, resulting in a constant torque. We have given a more detailed explanation of the components of the controller torques in Supplementary materials section S5.

5. The steady-state torque term is used to compensate for constant gravitational torque at the target posture, and the system will become a pendulum without gravity. But in the manuscript, gravity was just used in scaled model of posture task. To make all these experiments clearer, it’s better to just introduce gravity in scaled model of posture task. For others, the system will become much easier to just give the model without gravity. (Also, the introduction of PID controller is a little confusing, is there really a need to compare a steady state torque term with Integral term?)

We have considered gravity for both scaled models, the swing task and posture task. We use the steady state torque term only for the swing task, as the target position is +15°. We do not need a steady state torque in the posture task as the target position is at rest in the vertical posture.

We have considered an initial deadtime equal to the sensorimotor delay in the scaled models, during which the pendulum swings freely under gravity. The steady state torque term kicks in only when the neural controller detects the perturbation after the deadtime. Therefore, the behavior of the scaled swing task model cannot be reduced to a rotor (a pendulum without gravity). As a PD controller alone cannot correct for the steady-state error caused by gravity at the +15° position, we need either an integral term in the controller or the steady-state torque.

6. Line 462: “not reaching negative torque bound” is not an informative conclusion if the reason the negative torque limit is not reached is simply that the initial angle is positive. In other words, if I’m understanding correctly, the asymmetry in +/- clipping is due to the asymmetry in the input. This is fine, but perhaps worth reminding or clarifying (although I’m not sure I’m understanding the significance of this result).

Thank you for pointing this out. The torque profile of the normalized swing task feedback system has an initial acceleration phase with a peak value of 0.1617, followed by a slower braking phase which reaches a peak value of -0.0950 (Fig S4), due to the proportional and derivative terms dominating at different times in the response.

We stated that in the “middle” region of Fig 2, the response time increases slowly as only the positive torques are clipped, when compared to the “low” region where both positive and negative torques are clipped.

We have included the following lines to explain this in L458-460 in Results section 3.1:

“The torque curve has a fast acceleration phase which reaches a maximum of 0.1617, followed by a slower braking phase which reaches a peak of -0.0950 (Fig S4), due to the proportional and derivative terms dominating at different times in the response.*”*

Fig S4

7. Abstract (picky edits that I happened to notice): “full-size range” I don’t think you want the hyphen, it is the “full (size range)” not the “(full-size) range”. Also in the abstract, you don’t need “from literature” since you say in the same sentence it is published (redundant).

Thank you for pointing these out. We have corrected them.

8. Also, I don’t know whether the supporting section S2 (the Bode plot analysis of a linear feedback control system) is needed; it is highly tutorial and this is not a tutorial paper.

We acknowledge that the Bode plot section feels like a tutorial. We felt it would help our readers with a comparative biology background who would appreciate the control theory explanation. The explanation of how time delays and natural frequency (which are biological values) affect control systems (Kp and Kd) will be helpful to readers without a control theory background. The relationships between controller gains and time delay also inform how we decided normalization factors for the next section. We have added the following text to the end of section S2:

“These associations help inform our choice of non-dimensionalization factors in the next section.”

9. Also, my student with whom I co-reviewed has a Font error when opening the Supplementary materials on a Mac in Line 80, but it was fine for me; I recommend double-checking a final PDF of the supplementary information on multiple platforms to ensure font compatibility.

Thank you for pointing this out. We have checked for font errors and updated the supplementary materials document to address this issue.
